# Biomarker-driven drug repurposing for NAFLD-associated hepatocellular carcinoma using machine learning integrated ensemble feature selection

**DOI:** 10.3389/fbinf.2025.1522401

**Published:** 2025-04-17

**Authors:** Subhajit Ghosh, Sukhen Das Mandal, Subarna Thakur

**Affiliations:** ^1^ Department of Bioinformatics, University of North Bengal, Darjeeling, West Bengal, India; ^2^ Department of Computer Science and Engineering, Ghani Khan Choudhury Institute of Engineering and Technology (GKCIET), Malda, India

**Keywords:** NAFLD, HCC, machine learning, ensemble feature selection, drug repurposing, molecular docking

## Abstract

The incidence of non-alcoholic fatty liver disease (NAFLD), encompassing the more severe non-alcoholic steatohepatitis (NASH), is rising alongside the surges in diabetes and obesity. Increasing evidence indicates that NASH is responsible for a significant share of idiopathic hepatocellular carcinoma (HCC) cases, a fatal cancer with a 5-year survival rate below 22%. Biomarkers can facilitate early screening and monitoring of at-risk NAFLD/NASH patients and assist in identifying potential drug candidates for treatment. This study utilized an ensemble feature selection framework to analyze transcriptomic data, identifying biomarker genes associated with the stage-wise progression of NAFLD-related HCC. Seven machine learning algorithms were assessed for disease stage classification. Twelve feature selection methods including correlation-based techniques, mutual information-based methods, and embedded techniques were utilized to rank the top genes as features, through this approach, multiple feature selection methods were combined to yield more robust features important in this disease progression. Cox regression-based survival analysis was carried out to evaluate the biomarker potentiality of these genes. Furthermore, multiphase drug repurposing strategy and molecular docking were employed to identify potential drug candidates against these biomarkers. Among the seven machine learning models initially evaluated, DISCR resulted as the most accurate disease stage classifier. Ensemble feature selection identified ten top genes, among which eight were recognized as potential biomarkers based on survival analysis. These include genes ABAT, ABCB11, MBTPS1, and ZFP1 mostly involved in alanine and glutamate metabolism, butanoate metabolism, and ER protein processing. Through drug repurposing, 81 candidate drugs were found to be effective against these markers genes, with Diosmin, Esculin, Lapatinib, and Phenelzine as the best candidates screened through molecular docking and MMGBSA. The consensus derived from multiple methods enhances the accuracy of identifying relevant robust biomarkers for NAFLD-associated HCC. The use of these biomarkers in a multiphase drug repurposing strategy highlights potential therapeutic options for early intervention, which is essential to stop disease progression and improve outcomes.

## 1 Introduction

The alarming rise in the incidence of non-alcoholic fatty liver disease (NAFLD), triggered by obesity and the type 2 diabetes mellitus (T2D) epidemic, has increased the concerns within the healthcare system (Teng et al., 2023). NAFLD can progress to non-alcoholic steatohepatitis (NASH), liver fibrosis and cirrhosis, or even hepatocellular carcinoma (HCC), the most common form of primary liver cancer. HCC is associated with a poor prognosis, partly because it is often diagnosed at a late stage. Furthermore, the lack of approved pharmaceutical treatments specifically targeting NAFLD-induced HCC (Zhang and Yang, 2021) necessitates the urgent need for targeted therapeutic interventions. High-throughput omics technology has generated extensive gene expression datasets, allowing diverse new approaches to improve analysis and interpretation (Chen C. et al., 2023). The transition from NAFLD to HCC follows a prolonged timeline of 5–15 years, posing difficulties in conducting patient research (Straś et al., 2020). This challenge can be addressed by integrating data from various samples, though identifying key regulators of disease progression remains difficult. Conventional methods analyze disease stages separately, but merging these results often leads to issues like overfitting, technical noise, and reduced robustness (Posekany et al., 2011). These challenges can significantly impact the reliability and generalizability of the results. Machine learning-based feature selection has recently emerged as a solution to this problem. Recently, a machine learning (ML)-based approach has been applied to breast cancer prediction and classification by the detection of malignant cells using models like XGBoost, logistic regression, K-nearest neighbor, etc., (Chen H. et al., 2023). This kind of method holds great potential for early diagnosis of cancer. The results of the ML-based studies using imaging, such as lung cancer CT scans, showed promising results in identifying cancer subtypes (Nazir et al., 2023). Biomarker selection for early detection using gene expression data can be accomplished through robust feature selection methods, which have recently been applied in breast cancer (Sarkar et al., 2021), gastric cancer (Azari et al., 2023), lung and colon cancers (Talukder et al., 2022), etc., In gene expression analysis, effective feature selection techniques can pinpoint the most relevant and unique genes or molecular characteristics (Barrera et al., 2007), which facilitates the development of robust and easily interpretable gene signature models. Ensemble feature selection is a recently introduced approach, that enhances the robustness and accuracy of selected features by combining the results of multiple feature selection methods (Barrera et al., 2007; Bolón-Canedo and Alonso-Betanzos, 2019). In the last few years, this approach has been found to be effective in cancer gene expression data analysis, and in the identification of key genes as the most relevant features (Koul and Manvi, 2020; Khatun et al., 2023). In this study, after selecting and preprocessing microarray datasets, seven machine-learning techniques such as DISCR (Discriminant Analysis), NB (Naive Bayes), RF (Random Forest), DT (Decision Tree), KNN (K-Nearest Neighbors), SVM (Support Vector Machine), and ANN (Artificial Neural Network) were applied for disease stage classification. The method with the highest classification accuracy was selected using a 10-fold cross-validation for further analysis. Next, twelve feature selection strategies were utilized to identify the most significant genes. Among these twelve techniques, CIFE (Conditional Informative Feature Extraction), JMI(Joint Mutual Information), and MIM(Mutual information maximization) were based on mutual information, which selects features based on their relevance and dependency on the target variable, aiming to capture the most informative attributes for classification or analysis purposes (Gao et al., 2018). The Kendall Tau, Pearson, and Spearman methods selected the features based on their correlation or association with the target variable, focusing on measuring the strength and direction of the relationship between variables (El-Hashash and Shiekh, 2022). The other embedded methods, LASSO (Least Absolute Shrinkage and Selection Operator), Ridge, and gradient boosting utilize regularization techniques to penalize the complexity of the model, encouraging simpler models that generalize well to new data (Li Y. et al., 2022; Bhandari et al., 2022). These methods aim to prevent overfitting by constraining the coefficients of the features, thus promoting sparsity and improving the model’s interpretability and predictive performance. This study enhances feature selection, model regularization, and generalization by integrating various methods. This ensemble approach leverages diverse insights enhancing robustness and stability while revealing complex data patterns. (Castellanos-Garzón et al., 2017). This approach of feature selection helps in identifying the key genes as features that are subsequently evaluated for their prognostic potential as biomarkers in HCC applying the Cox proportional hazards model (Mohammed et al., 2021). Furthermore, it incorporates a biomarker-driven drug repurposing approach using identified biomarker genes as targets to screen out suitable drug candidates from the library of existing drugs. Drug repurposing has gained attention identification of novel uses of existing drugs, either through new combinations or in the treatment of different diseases (Krishnamurthy et al., 2022). Potential drugs were identified using connectivity map analysis, text mining, drug-gene interaction data, etc. These drug candidates were further assessed through molecular docking to evaluate their binding affinity with target proteins and to explore the therapeutic potential of these existing drugs against new targets.

## 2 Materials and methods

### 2.1 Data collection and preparation

**FIGURE 1:**
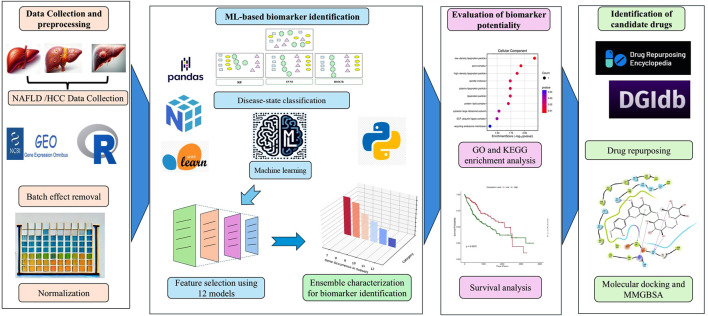
Schematic diagram depicting methodology.

The microarray data and clinical information for Control, Healthy obese, NAFLD, NASH, and HCC samples were obtained from the NCBI Gene Expression Omnibus (GEO) (Barrett et al., 2013) and Array Express (Parkinson et al., 2007) database. This dataset comprised a total of 132 samples with GEO accession numbers: GSE48452 with platform ID - GPL11532 (Affymetrix Human Gene 1.1 ST Array); GSE25097 with platform ID - GPL10687 (Rosetta/Merck Human RSTA Affymetrix 1.0 microarray). Details of datasets and respective sample information were provided as Supplementary Material S1. Following the merging of data, the R package “imputeTS” (version 3.3) (Moritz and Bartz-Beielstein, 2017) was utilized to impute missing values. This imputation process was only carried out on less than 5% of randomly missing values. Afterward, the “Limma” (version 3.57.3) package (Ritchie et al., 2015) was utilized to remove batch effects specific to each study, and data normalization was performed utilizing the robust multichip averaging (RMA) method (Bioconductor, 2023). Figure 1 presents a schematic diagram representing the methodology.

### 2.2 Disease state classification using machine learning

The entire dataset, encompassing samples from various disease stages ranging from NAFLD to HCC, is utilized to identify a suitable machine-learning technique with the primary objective of effectively categorizing the data into distinct groups based on disease stages. Each of the following machine learning (ML) models is applied individually to the entire pre-processed dataset using 10-fold cross-validation: DISCR (Discriminant Analysis), NB (Naive Bayes), RF (Random Forest), DT (Decision Tree), KNN (K-Nearest Neighbors), SVM (Support Vector Machine), and ANN (Artificial Neural Network). This procedure guarantees a thorough assessment and reduces the risk of overfitting by training and verifying the models on distinct subsets of the data. Through this screening, the ML technique with the highest accuracy values and other pertinent performance indicators is chosen for further investigation.

### 2.3 Ensemble feature selection approach for identification of genes involved in disease progression

Twelve different feature selection methods are applied to the entire dataset. Each of these methods individually selects subsets of genes interactively, comprising varying quantities, such as the top 20, 30, 40, 50, and beyond. Subsequently, each subset generated by the different methods is utilized in the chosen machine learning technique, employing 10-fold cross-validation to calculate the classification accuracy. This guarantees that the performance of the chosen characteristics is comprehensively assessed. Furthermore, each feature selection method yields a subset of genes based on the highest level of accuracy attained. This technique enables the identification of the most pertinent genes that make a major contribution to classification, ensuring a strong and dependable selection of features. Among the feature selection techniques used, the ReliefF algorithm evaluates each feature’s significance based on its ability to distinguish between similar cases. It iteratively selects a sample, comparing it with the nearest samples from both the same and different classes. Features that effectively differentiate between classes receive higher weights, while those that distinguish within the same class receive lower weights (Urbanowicz et al., 2018). The process is repeated across multiple instances to reliably estimate feature importance, making ReliefF effective for noisy, multi-class datasets. The basic equation for updating the feature weights in ReliefF is:

$$W\left[A\right]=W\left[A\right]-\frac{1}{m}\sum_{i=1}^{k}\text{diff}\left(A,R,{\text{Hit}}_{i}\right)+\frac{1}{m}\sum_{C\neq \text{class}\left(R\right)}\frac{P\left(C\right)}{1-P\left(\text{class}\left(R\right)\right)}\sum_{i=1}^{k}\text{diff}\left(A,R,{\text{Miss}}_{i}^{C}\right)$$

Where,

W [A] This represents the updated weight or importance score of feature AAA.

$W\left[A\right]$ on the right-hand side: This term indicates the current weight of feature A.

$\frac{1}{m}\sum_{i=1}^{k}\text{diff}\left(A,R,{\text{Hit}}_{i}\right)$ This part calculates the average difference in feature A values between the Randomly decided on instance R and its K nearest neighbors that belong to the same class as R (denoted as ${\text{Hit}}_{i}$ ).

Cramér’s V is a filter-based technique that measures the correlation between two nominal variables. It involves creating a contingency table to compute the Chi-Squared statistic, which quantifies the strength of the association between the variables (Kearney, 2017). To account for the bias, the Chi-Squared estimate is reformulated as a Phi-Squared value. The degrees of freedom are subtracted from both the row and column counts to obtain the denominator of the Cramér’s V formula. Taking the square root of the quotient resulting from dividing the corrected Phi-Squared value by the denominator yields Cramér’s V in the range from 0 for no association up to 1 for the perfect association. This measure helps determine categorical data as it offers an idea of the strength with which variables are correlated without the assumption of any linear relation.

Chi-Squared Statistic $\left(\chi 2\right)$ :

$$\chi^{2}=\sum \frac{{\left(O_{i}-E_{i}\right)}^{2}}{E_{i}}$$

where $O_{i}$ is the observed frequency and $E_{i}$ is the expected frequency.

Phi-Squared ( $\phi^{2}$ ):

$$\phi^{2}=\frac{\chi^{2}}{n}$$

where n is the total number of observations.

Corrected Phi-Squared $\left(\phi_{\text{corr}}^{2}\right)$ :

$$\phi_{\text{corr}}^{2}=\max\left(0,\phi^{2}-\frac{\left(k-1\right)\left(r-1\right)}{n-1}\right)$$

where r and k are the number of rows and columns in the contingency table.

Corrected Row and Column Counts

$$r_{\text{corr}}=r-\frac{{\left(r-1\right)}^{2}}{n-1}$$

$$k_{\text{corr}}=k-\frac{{\left(k-1\right)}^{2}}{n-1}$$

Denominator:

$$\text{denominator}=\min\left(k_{\text{corr}}-1,r_{\text{corr}}-1\right)$$

Cramér’s V:

$$V=\sqrt{\frac{\phi_{\text{corr}}^{2}}{\text{denominator}}}$$

Pearson’s correlation coefficient (r) quantifies the strength and direction of a linear relationship between two variables (Nasir et al., 2020), ranging from −1 to +1. A value of +1 indicates a perfect positive correlation, where both variables increase together; −1 signifies a perfect negative correlation, where one variable increases as the other decreases; and 0 denotes no linear correlation.

Pearson’s correlation coefficient ( r ) is defined as:

$$r=\frac{n\left(\sum xy\right)-\left(\sum x\right)\left(\sum y\right)}{\sqrt{\left[n\sum x^{2}-{\left(\sum x\right)}^{2}\right]\left[n\sum y^{2}-{\left(\sum y\right)}^{2}\right]}}$$

Where:

• n is the number of data points.
• x and y are the two variables being compared.
• ∑xy is the sum of the product of x and y .
• ∑x is the sum of x .
• ∑y is the sum of y .
• $\sum x^{2}$ is the sum of the squares of x .
• $\sum y^{2}$ is the sum of the squares of y .

Kendall’s Tau is a non-parametric measure that quantifies the ordinal correlation between two variables (Valencia et al., 2019), focusing on the direction and magnitude of their association. While Pearson’s correlation coefficient measures only linear relationships, Kendall’s Tau is a measure that is very useful in cases where observations do not meet the assumptions of normality or linearity. It counts concordant and discordant pairs of observations for its calculation. Values range from −1 (perfect inverse correlation) up to +1 for perfect direct correlation. A value of 0 indicates no association.

Kendall’s Tau $\left(\tau \right)$ can be defined as:

$$\tau =\frac{\left(\text{Number}\;\text{of}\;\text{concordant}\;\text{pairs}\right)-\left(\text{Number}\;\text{of}\;\text{discordant}\;\text{pairs}\right)}{\left(\begin{array}{l} n\\ 2 \end{array}\right)}$$

Where:

• Concordant pairs: For any two pairs of observations $\left(x_{i},y_{i}\right)$ and $\left(x_{j},y_{j}\right)$ the pairs are concordant if the order of the elements is the same, i.e., $\left(x_{i}<x_{j}\text{ and }y_{i}<y_{j}\right)\text{ or }\left(x_{i}>x_{j}\text{ and }y_{i}>y_{j}\right).$

• Discordant pairs: For any two pairs of observations $\left(x_{i},y_{i}\right)$ and $\left(x_{j},y_{j}\right)$ , the pairs are discordant if the order of the elements is opposite, i.e., $\left(x_{i}<x_{j}\text{ and }y_{i}>y_{j}\right)\text{ or }\left(x_{i}>x_{j}\text{ and }y_{i}<y_{j}\right).$

• $\left(\begin{array}{l} n\\ 2 \end{array}\right)$ is the total number of pairs, calculated as $\frac{n\left(n-1\right)}{2}.$

Another method, Spearman’s rank correlation coefficient (ρ) is a statistical measure that quantifies the degree and direction of the relationship between two variables that have been ranked (Schober and Schwarte, 2018). It measures how well the relationship between two variables can be represented as a monotonic function. This means that the Spearman rank correlation varies from Pearson’s correlation, as it considers the variables’ ranks while trying to figure out their relationship and not the variable’s actual values. Hence, it is applicable in assessing ordinal data that deviate from the assumptions of linearity and normalcy.

Spearman’s rank correlation coefficient $\left(\rho \right)$ is defined as:

$$\rho =1-\frac{6\sum d_{i}^{2}}{n\left(n^{2}-1\right)}$$

Where:

• n is the number of data points.
• $d_{i}$ is the difference between the ranks of corresponding variables.

On the other hand, Conditional Infomax Feature Extraction (CIFE) selects features by maximizing a score J(S) (Lin and Tang, 2006). Features are incrementally added to the set *S* based on their significance and redundancy relative to already chosen features. In this way, the algorithm continues till it gets some pre-defined features or threshold scores. CIFE intends to offer an informative feature set that is concise in length, by managing relevance and redundancy towards better facilitation of subsequent ML models.

The CIFE can be expressed as

$$J\left(S\right)=\sum_{X_{i}\in S}I\left(X_{i};Y\right)-\beta \sum_{X_{i},X_{j}\in S}I\left(X_{i};X_{j}|Y\right)$$

Where:

• S is the set of selected features.
• $I\left(Xi;Y\right)$ is the mutual information between feature $X_{i}$ and the target variable Y .
• $I\left(Xi;Xj|Y\right)$ is the conditional mutual information between features Xi and Xj given the target variable Y .
• β is a parameter that controls the trade-off between relevance and redundancy.

Among mutual information-based feature selection methods, the Joint Mutual Information (JMI) criterion is used to identify the most relevant features for a specific task. JMI selects features based on their high mutual information with the target variable while considering the combined information among selected features (Bennasar et al., 2015). This way of approaching features ensures that they are individually relevant and also collectively informative to reduce redundancy while enhancing the predictive capability of the model. JMI helps build an efficient and effective feature set, resulting in balancing relevance with redundancy.

The JMI criterion can be defined as:

$$J\left(X_{i};Y,S\right)=I\left(X_{i};Y\right)+\sum_{X_{j}\in S}I\left(X_{i};X_{j}|Y\right)$$

Where:

• $I\left(Xi;Y\right)$ is the mutual information between feature Xi and the target variable Y.

• $I\left(Xi;Xj|Y\right)$ is the conditional mutual information between feature Xi and an already-selected feature Xj given the target variable Y .
• S is the set of already selected features.

The goal is to maximize $J\left(Xi;Y,S\right)$ to select features that contribute the most information about the target variable while considering the redundancy with already selected features.

Another mutual information-based method, The Maximum Relevance (MIM) criterion is a technique for selecting features that have the most mutual information with the target variable (Che et al., 2017). The main objective of MIM is to identify those attributes with the highest relevance to predict the outcome, such that it improves the performance of the machine learning models. This technique lessens the dimensionality of the data without losing the most informative attributes. MIM especially comes in handy in applications where the dataset comprises several features, and the identification of the most relevant ones can greatly enhance model efficiency and accuracy. The MIM criterion can be mathematically defined as:

$$MIM\left(Xi,Y\right)=I\left(Xi;Y\right)$$

Where:

• $I\left(Xi;\;Y\right)$ is the mutual information between feature Xi ​ and the target variable Y .

Mutual information $I\left(X;\;Y\right)$ measures the amount of information obtained about one variable through another variable and is defined as:

$$I\left(X_{i};Y\right)=\sum_{x_{i}\in X_{i}}\sum_{y\in Y}p\left(x_{i},y\right)\log\frac{p\left(x_{i},y\right)}{p\left(x_{i}\right)p\left(y\right)}$$

where:

• $p\left(x,y\right)$ is the joint probability distribution function of X and Y .
• $p\left(x\right)$ and $p\left(y\right)$ are the marginal probability distribution functions of X and Y respectively.

Normalized Mutual Information Feature Selection (NMIFS) is a feature selection technique that aims to find and rank features based on their normalized mutual information in relation to the target variable (Estévez et al., 2009). Mutual information quantifies interdependence between variables, showing how much one can inform about another. By standardizing this metric, NMIFS accounts for variable scales and distributions, helping to identify the most informative features for predicting the target variable and enhancing the effectiveness and precision of machine learning models. The NMIFS score for a feature Xi with respect to the target variable Y can be defined as:

$$\text{NMIFS}\left(X_{i},Y\right)=\frac{I\left(X_{i};Y\right)}{H\left(X_{i}\right)+H\left(Y\right)}$$

Where:

• $I\left(Xi;\;Y\right)$ is the mutual information between feature Xi and the target variable Y .
• $H\left(Xi\right)$ is the entropy of the feature $\left(Xi\right)$ .
• $H\left(Y\right)$ is the entropy of the target variable Y .

Lasso, or Least Absolute Shrinkage and Selection Operator, is a regularization technique in linear regression that enhances feature selection and controls model complexity (Muthukrishnan and Rohini, 2017). By adding a penalty based on the absolute values of coefficients, Lasso reduces the coefficients of less important features to zero, promoting simpler models and avoiding overfitting. It is widely used in data science and machine learning to streamline models and focus on the most relevant features. The objective function minimized by Lasso is given by:

$$\min_{\beta }\left\{\frac{1}{2N}\sum_{i=1}^{N}{\left(y_{i}-\sum_{j=1}^{p}x_{ij}\beta_{j}\right)}^{2}+\alpha \sum_{j=1}^{p}\left|\beta_{j}\right|\right\}$$

Where:

• N is the number of samples.
• p is the number of features.
• yi is the target variable for sample i .
• xij is the value of feature j for sample i .
• βj is the coefficient of feature j .
• α is the regularization parameter that controls the strength of the penalty term $\sum_{j=1}^{p}\left|\beta_{j}\right|.$

Ridge regression is a regularization method employed in linear regression models to mitigate overfitting and enhance generalization (Paul and Drineas, 2016). Ridge regression employs a $\left(L_{2}\right)$ penalty on the regression coefficients, in contrast to Lasso regression which utilizes a $\left(L_{1}\right)$ penalty. The penalty term is determined by a regularization parameter $\left(\alpha \right)$ , which determines the trade-off between accurately fitting the data and punishing big coefficients.

$$\min_{\beta }\left\{\frac{1}{2N}\sum_{i=1}^{N}{\left(y_{i}-\sum_{j=1}^{p}x_{ij}\beta_{j}\right)}^{2}+\alpha \sum_{j=1}^{p}\beta_{j}^{2}\right\}$$

N is the number of samples.

p is the number of features.

yi is the target variable for sample i .

xij is the value of feature j e for sample i .

$\beta_{j}$ is the coefficient of feature j .

α is the regularization parameter that controls the strength of the penalty term $\sum_{j=1}^{p}\beta_{j}^{2}$

Gradient Boosting is an advanced ensemble learning method that iteratively combines decision trees. Unlike Random Forests, which build trees independently, Gradient Boosting constructs trees sequentially, with each tree correcting errors made by its predecessors (Otchere et al., 2022). It uses feature importance to identify significant features, revealing how much each feature contributes to accurate predictions. High feature importances allow for more contribution toward the overall impact of model performance while allowing some form of implicit feature selection, especially with complex datasets, making the model perform better and be more interpretable. The method includes several steps, such as -

Initialization of the model with a Constant:

Initialization of the model with a constant value, typically the mean of the target values for a regression problem. This can be represented as: $F_{0}\left(x\right)=\arg\min_{c}\sum_{i=1}^{n}L\left(y_{i},c\right)$

Where $L\left(y,c\right)$ is the loss function, $y_{i}$ are the true target values, and c is a constant.

Iterative tree building:

For m=1 to M (where M is the number of trees to be built):

a. Computing the Pseudo-Residuals: $r_{im}=-\left[\frac{\partial L\left(y_{i},F_{m-1}\left(x_{i}\right)\right)}{\partial F_{m-1}\left(x_{i}\right)}\right]$

where $r_{im}$ are the pseudo-residuals for each instance i at iteration m , and $F_{m-1}\left(x\right)$ is the prediction from the previous iteration.

b. Fitting a Base Learner: Fit a decision tree (base learner) $h_{m}\left(x\right)$

c. to the pseudo-residuals $r_{im}:h_{m}=\arg\min_{h}\sum_{i=1}^{n}{\left(r_{im}-h\left(x_{i}\right)\right)}^{2}$

d. Updating the Model: Updating the model by adding the newly fitted tree, scaled by a learning rate $\eta :F_{m}\left(x\right)=F_{m-1}\left(x\right)+\eta h_{m}\left(x\right)$

Combining the Trees:

The final model $F\left(x\right)$ after M iterations is: $F\left(x\right)=F_{0}\left(x\right)+\sum_{m=1}^{M}\eta h_{m}\left(x\right)$

Next, Feature selection in Gradient Boosting is typically achieved by examining feature importances derived from the model. Each feature’s importance is calculated based on its contribution to reducing the model’s prediction error. Feature importance can be computed as follows:

• Calculating the total reduction in the loss function due to splits involving each feature across all trees.
• Aggregating these reductions to assign an importance score to each feature.

Mathematically, the importance of the feature j can be represented as:

$$\text{Importance}\left(j\right)=\sum_{m=1}^{M}\sum_{t=1}^{T_{m}}\Delta L_{j,mt}$$

Where, $T_{m}$ is the number of nodes in the tree, and is the reduction in the loss function due to the split on feature j at node t in tree m .

### 2.4 Categorization of features using an ensemble of different subsets of features

The genes obtained from different feature selection approaches are used as subsets. These subsets were then used to create an ensemble by categorizing the genes by assigning values 12 to 1. A gene is classified as 12 if it appears in all twelve subsets, 11 if it is present in eleven of the twelve subsets, and so on. Subsequently, genes that fell into at least 6 subsets were selected for Cox regression-based survival analysis. This decision guarantees that a minimum of 50% of the feature selection methods will support the inclusion of these genes, hence strengthening the reliability subsequent to analysis.

### 2.5 GO and KEGG enrichment analysis

The ensemble approach yielded a consolidated list of genes, then annotated using DAVID tool version 6.8 (https://david.ncifcrf.gov/) (Sherman et al., 2022). The criteria for conducting Gene Ontology (GO) and KEGG pathway enrichment analysis were set as a p-value below 0.05 and a false discovery rate (FDR) below 0.05. The GO enrichment analysis was utilized to ascertain the biological activities of these genes. GO ontologies are divided into three categories: molecular function (MF), cellular component (CC), and biological process (BP). The KEGG pathway enrichment analysis identified metabolic pathways that showed a significant enrichment of genes, as compared to the total genome background. The SRPlot online toolkit (http://www.bioinformatics.com.cn/srplot) (Tang et al., 2023) was used to display the findings of the Gene Ontology (GO) and Kyoto Encyclopedia of Genes and Genomes (KEGG) pathway enrichment studies.

### 2.6 Survival analysis

Survival analysis was performed using the TCGA-LIHC (Liver Hepatocellular Carcinoma) dataset to investigate the association between the expression level of the genes and overall survival. First, clinical data and gene expression data were retrieved from the TCGA-LIHC cohort using the “TCGAbiolinks” package (Colaprico et al., 2016). The gene expression data is preprocessed, and a Variance Stabilizing Transformation (VST) is applied using DESeq2 (Love et al., 2014). The expression levels of the genes were extracted, and a median value was calculated to stratify the samples into “HIGH” and “LOW” expression groups. Next, the clinical data is merged with the gene expression data, and a Cox proportional hazards regression model is fitted to estimate the hazard ratio (HR) and its 95% confidence interval (CI) using the survival package (Therneau, 2021). Finally, a Kaplan-Meier survival curve is plotted using the “survminer” package (Pawar et al., 2022).

### 2.7 Screening of possible drug candidates for repurposing

The biomarkers identified through survival analysis were used as input in two separate databases i.e., DGIdb (The Drug Gene Interaction Database) (Cannon et al., 2024) and Drug repurposing encyclopedia (Li et al., 2023). Additionally, clue.io COMMAND app web tool (Xie et al., 2022) was utilized to screen potential drug candidates. The DGIdb database uses a combination of expert curation and text-mining approaches to mine drug-gene interactions mined from DrugBank, PharmGKB, ChEMBL, Drug Target Commons, and others. On the other hand, The Drug Repurposing Encyclopedia utilizes the Molecular Signatures Database (MSigDB, https://www.gsea-msigdb.org/gsea/msigdb/), and consensus drug profiles from DREIMT (http://www.dreimt.org/), which are derived from the Connectivity Map (CMap) LINCS gene expression dataset (https://clue.io/). The command app also utilizes connectivity map (CMap) analysis to screen possible drugs.

### 2.8 Molecular docking and MMGBSA analysis

#### 2.8.1 Structure retrieval

The potential drugs screened through drug repurposing were used as ligands in the docking analysis against the targets implicated in disease progression. The chemical structures of the drugs were obtained from the PubChem database (https://pubchem.ncbi.nlm.nih.gov/) (Wang et al., 2009). The structures of the targets, namely, ABAT (PDB ID: 1OHW), ABCB11 (PDB ID: 6LRO), C8B (PDB ID: 3OJY), and FBX23 (PDB ID: 416J), were obtained from the RCSB database (https://www.rcsb.org/) (Deshpande et al., 2005) in PDB format. The RCSB-PDB database (https://www.rcsb.org/) (Deshpande et al., 2005) lacked monomeric structures for the proteins APOF, CENPV, MBTPS1, and ZFP1. Therefore, the protein sequences were obtained from the NCBI database, and a BLAST search was conducted in the SWISS-MODEL template library to identify structurally comparable homologous structures. The structures exhibiting similarity were subsequently acquired via the SWISS-MODEL tool (Kiefer et al., 2009).

#### 2.8.2 Protein and ligand preparation

The proteins were prepared using the Schrödinger software (Maestro Version 12.5.139, Schrödinger, LLC, New York) with the OPLS3 force field (Harder et al., 2016) to ensure precise depiction. Similarly, the ligands were produced using the OPLS3 force field in LigPrep (Maestro Version 12.5.139, Schrödinger, LLC, New York). This preparatory step aimed to ensure that the docking analysis was consistent and reliable.

#### 2.8.3 Docking procedure

Before docking analysis, receptor grids were created for each protein using Glide (Maestro Version 12.5.139, Schrödinger, LLC, New York) to ensure precise accommodation. The grids were constructed with accurate coordinates based on binding pocket predictions from PrankWeb (https://prankweb.cz/) (Jendele et al., 2019). After generating the grids, the docking scores were calculated systematically using Glide’s Extra-precision (XP) docking model. The scores provided measurable data on the binding affinities between each ligand and its respective protein target.

#### 2.8.4 Binding energy calculations using Prime/MM GBSA analysis

The binding free energies of the protein-ligand complex are evaluated using the Molecular Mechanics-Generalized Born Surface Area (MM-GBSA) approach, implemented in the Prime module of the Schrödinger suite (Maestro Version 12.5.139, Schrödinger, LLC, New York). The calculations utilize the OPLS 2005 force field and the VSGB solvation model for accurate energy estimations.

## 3 Results

### 3.1 Key features selected by the ensemble feature selection approach

**FIGURE 2:**
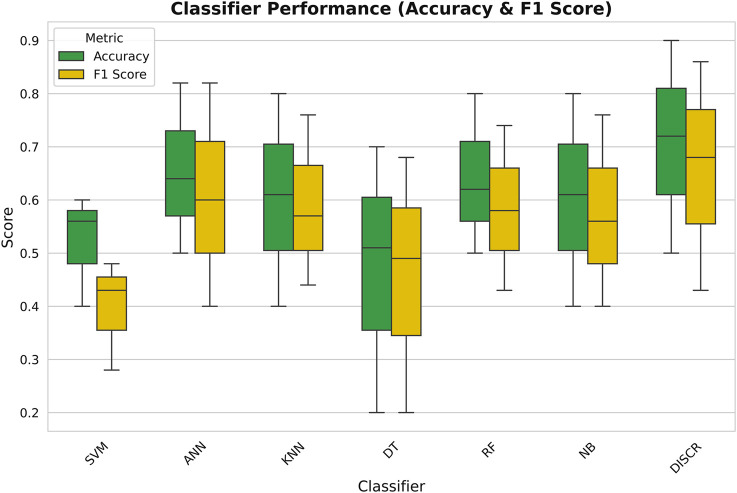
Box plot comparing the performance of different classifiers in terms of Accuracy (green) and F1 Score (yellow).

After data preparation, seven well-known machine learning techniques such as DISCR, NB, RF, DT, KNN, SVM, and ANN, were tested on the entire dataset. Figure 2 illustrates the accuracy scores of these machine learning techniques, each conducted independently on the whole preprocessed dataset using 10-fold cross-validation. Based on the accuracy values, DISCR outperformed other machine learning techniques, achieving the highest accuracy score of 0.90.

**FIGURE 3:**
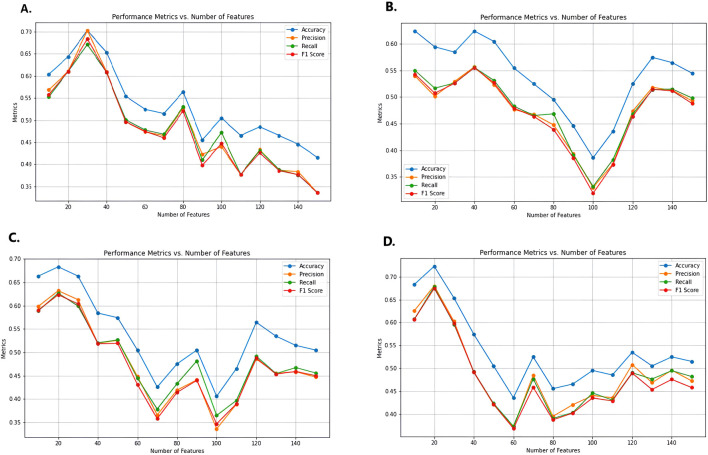
Performance metrics for different feature selection models, **(A)** Relief F, **(B)** Cammers V, **(C)** Kendall Tau, and **(D)** Pearson Correlation. Metrics for the rest of the feature selection models (E-L) were provided as supplementary file 2. Here X-axis represents the numbers of features, and the Y-axis represents performance metrics.

After selecting DISCR as the optimal classification method, twelve feature selection models were applied to identify the most significant gene subsets. These models included ReliefF, Cramér’s V, Kendall’s Tau, Pearson’s correlation, Spearman’s correlation coefficient, CIFE, JMI, MIM, NMIFS, LASSO, RIDGE, and Gradient Boosting. Iteratively, each of the twelve methods separately selects different subsets of genes like top 30, 40, 50, and so on. DISCR with 10-fold cross-validation is used for each subset of the different methods separately to check the classification accuracy. The accuracy details for different subsets of the genes, such as 30, 40, 50 to 150 genes, are reported in Table 1. Out of all the filter-based strategies, Relief F achieved the greatest accuracy of 0.70297 using a set of 30 characteristics. Similarly, the accuracy reached by Cramér’s V, Kendall Tau, Pearson’s correlation, and Spearman’s correlation coefficient was 0.623762, 0.683168, 0.722772, and 0.673267, respectively. These accuracies were obtained using 40, 20, 20, and 10 features. Among the approaches that use mutual information, CIFE, JMI, MIM, and NMIFS achieved accuracies of 0.643564, 0.752475, 0.475248, and 0.712871 using 40, 30, 70, and 20 features, respectively. Within the set of embedded methods, the LASSO algorithm achieved the highest accuracy score of 1 while using 40 features. Ridge and elastic-net algorithms achieved an accuracy of 0.752475 and 0.712871, respectively, using a total of 30 features. Figure 3 illustrates the performance matrices of different feature selection models (A-D) (Performance metrics for different feature selection models (E-L) were provided as Supplementary Material S2).

**TABLE 1:** Classification accuracy values of DISCR using twelve separate feature selection methods.

Number of features	Relief F	Cammers V	Kendall tau	Pearson correlation coefficient	Spearman’s rank correlation coefficient	CIFE	JMI	MIM	NMIFS	LASSO	RIDGE	Gradient boosting
10	0.603	0.623	0.663	0.683	0.673	0.603	0.663	0.435	0.663	0.762	0.663	0.722
20	0.643	0.594	0.683	0.722	0.653	0.643	0.712	0.376	0.712	0.831	0.742	0.693
30	0.702	0.584	0.663	0.653	0.653	0.613	0.752	0.465	0.663	0.891	0.752	0.712
40	0.653	0.623	0.584	0.574	0.633	0.643	0.683	0.465	0.673	1	0.732	0.712
50	0.554	0.603	0.574	0.504	0.574	0.564	0.683	0.415	0.683	1	0.673	0.663
60	0.524	0.554	0.504	0.435	0.534	0.495	0.584	0.455	0.653	0.99	0.574	0.613
70	0.514	0.524	0.425	0.524	0.485	0.504	0.554	0.475	0.574	0.891	0.524	0.475
80	0.564	0.495	0.475	0.455	0.475	0.495	0.524	0.475	0.603	0.643	0.564	0.524
90	0.455	0.445	0.504	0.465	0.485	0.504	0.504	0.435	0.603	0.673	0.603	0.405
100	0.504	0.386	0.405	0.495	0.504	0.534	0.524	0.465	0.554	0.504	0.554	0.504
110	0.465	0.435	0.465	0.485	0.495	0.504	0.504	0.435	0.534	0.504	0.594	0.524
120	0.485	0.524	0.564	0.534	0.524	0.485	0.495	0.455	0.524	0.504	0.623	0.455
130	0.465	0.574	0.534	0.504	0.495	0.435	0.475	0.435	0.475	0.504	0.594	0.564
140	0.445	0.564	0.514	0.524	0.534	0.504	0.495	0.425	0.504	0.504	0.574	0.475
150	0.415	0.544	0.504	0.514	0.564	0.514	0.495	0.415	0.445	0.504	0.594	0.455

The subsets of genes obtained from different feature selection approaches are subsequently merged to create an ensemble set of genes. These genes are then categorized from 12 to 1 using a consensus approach with those scoring at least 6 being considered as most important features. Through this ensemble approach a set of ten genes were identified. These genes include *C8B* (Complement C8 Beta Chain), *APOF* (Apolipoprotein F), *FBXL3* (F-Box and Leucine Rich Repeat Protein 3), *ABAT* (4-Aminobutyrate Aminotransferase), *ZFP1* (ZFP1 Zinc Finger Protein), *MBTPS1*(Membrane Bound Transcription Factor Peptidase, Site 1), *CENPV* (Centromere Protein V), *METTL23* (Methyltransferase 23, Arginine), *RPL9* (Ribosomal Protein L9), and *ABCB11* (ATP Binding Cassette Subfamily B Member 11).

### 3.2 Evaluating the biomarker potential through survival analysis

**FIGURE 4:**
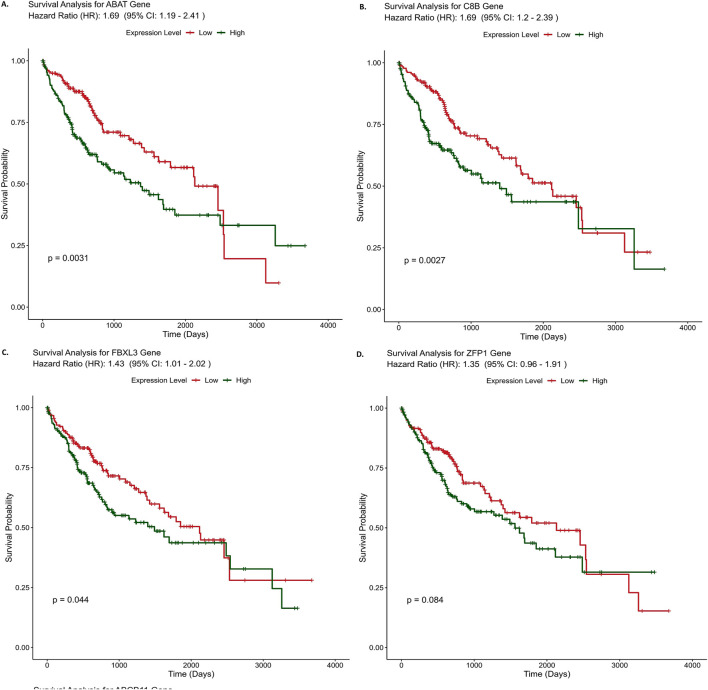
Survival curve analysis results of three hub genes **(A)**- ABAT, **(B)**- C8B, **(C)**- FBXL3 and **(D)**- ZFP1. X-axis represents time in months and Y-axis represents denotes survival probability, survival curve for other genes (E-J) are provided in supplementary file 3.

Kaplan-Meier survival analysis was used to evaluate the predicted survival probability over time in liver cancer patients, comparing normal and HCC samples based on the expression levels of key genes identified through an ensemble approach. Out of the ten identified genes, eight genes were found to have the worst overall survival rate for HCC. The identified genes were *ABAT* (HR: 1.69), *C8B* (HR: 1.69), *FBXL3* (HR: 1.43), *ZFP1* (HR: 1.35), *ABCB11*(HR: 1.33), *MBTPS1*(HR: 1.21), *CENPV* (HR: 1.2) and *APOF* (HR: 1.18). In the case of *ABAT* gene, the average hazard ratio stands at 1.69. This suggests that individuals with high gene expression face a 69% elevated risk of death compared to those with low gene expression, regardless of the circumstance as depicted in Figure 4. Similarly, for *C8B* and *FBXL3*, high expression corresponds to a 69% and 43% increased risk of death, respectively, in comparison to scenarios with low expression of these genes. HR value greater than 1 indicates a higher hazard rate in the reference group compared to the comparison group, suggesting that genes with higher expression in the comparison group are associated with an increased risk of death. Therefore, these genes could serve as predictive markers for poor prognosis. Survival analysis results of the rest of the genes are provided in Supplementary Material S3.

### 3.3 GO term enrichment analyses and KEGG pathway analyses

**FIGURE 5:**
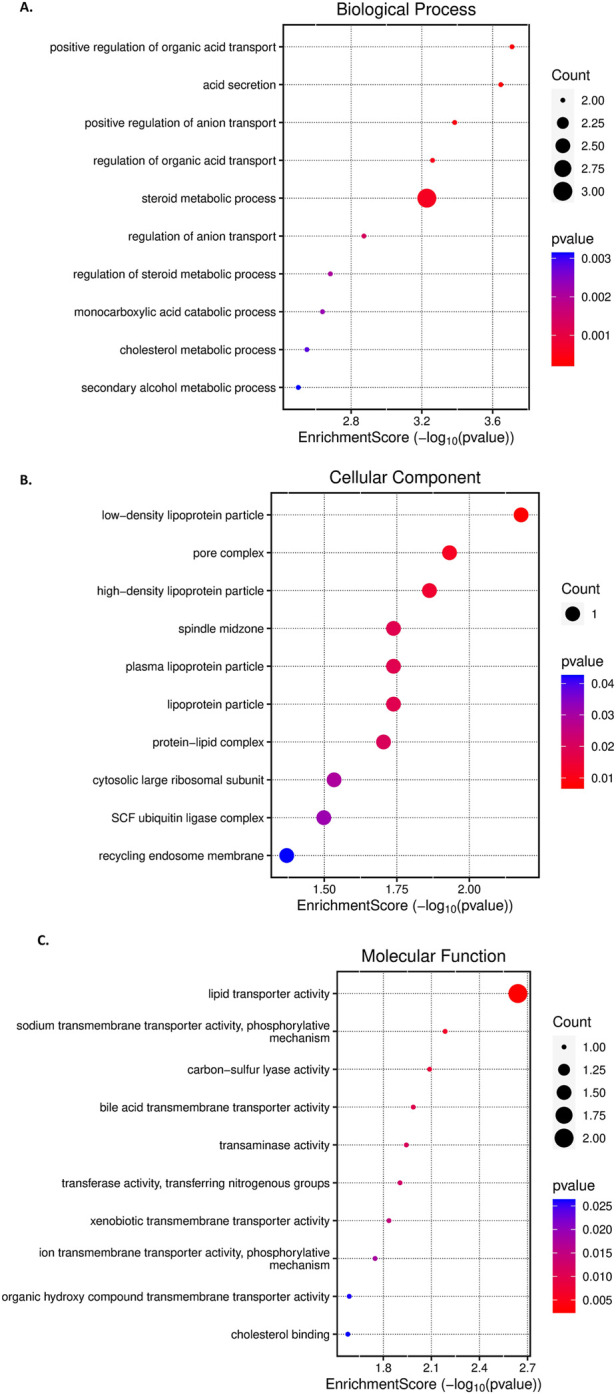
The results of the Gene Ontology (GO) enrichment analysis, visualized through a bubble chart, **(A)**-GO terms related to Biological Processes, **(B)**-GO terms associated with Cellular Components, and **(C)**-GO terms related to Molecular Functions. Each bubble on the Y-axis represents a different GO term, while the X-axis displays the enrichment score. The size of each bubble corresponds to the gene counts associated with the term. The colour of the bubbles reflects the P-value for each GO term, with the intensity of red indicating higher corrected P-values.

The gene ontology analysis using the ten identified genes from feature selection and based on the selected identifier - “OFFICIAL GENE SYMBOL,” and p-value < 0.05 and FDR <0.05 cut-offs yielded significant GO terms, depicted in Figure 5, for the marker genes. The enriched GO terms related to biological processes found to be negative regulation of gamma-aminobutyric acid secretion (GO:0014053), histone H3-R17 methylation (GO:0034971), gamma-aminobutyric acid biosynthetic process (GO:0009449), positive regulation of catecholamine metabolic process (GO:0045915), regulation of cholesterol biosynthetic process (GO:0045540), regulation of sterol biosynthetic process (GO:0106118), etc. The results suggest the involvement of metabolism and epigenetic processes, indicating dynamic changes in the disease progression. The top GO terms associated with cellular components terms identified as membrane attack complex (GO:0005579), low-density lipoprotein particle (GO:0034362), pore complex (GO:0046930), plasma lipoprotein particle (GO:0034358), etc; suggesting the involvement of membrane structure changes, lipid transport, etc, that are key characteristics of changes due to lipid accumulation and oxidative damage in hepatic cells in cellular dysfunction. The GO terms associated with molecular functions are histone-arginine N-methyltransferase activity (GO:0008469), carbon-sulfur lyase activity (GO:0016846), cholesterol binding (GO:0015485), pyridoxal phosphate binding (GO:0030170), etc., indicating lipid interaction and metabolic processes as important factors in this disease progression.

**FIGURE 6:**
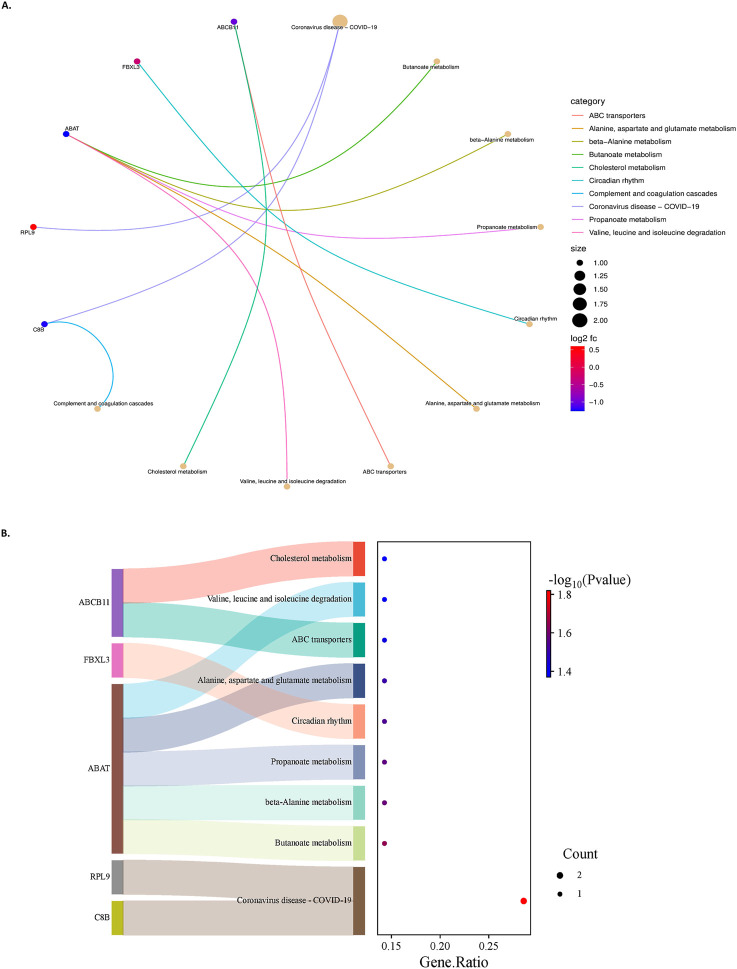
**(A)**- A chord plot represents pathways and genes’ relationship. The size of the pathway bubbles increases with the number of connected genes. **(B)**- The left side displays a Sankey plot, illustrating the genes within each pathway. The right side features a dot plot, where the dot sizes represent the number of genes, and the dot colours indicate P values.

The genes were also subjected to KEGG pathway analysis to determine their association with their corresponding biological pathways. A total of 13 pathways-related KEGG terms were obtained from the database. The criteria of p-value less than 0.05 and FDR less than 0.05 were used for analysis. The top enriched pathways and the respective gene counts are depicted in Figure 6. The KEGG analysis results showed that the genes are enriched in Butanoate metabolism (hsa00650), Propanoate metabolism (hsa00640), Alanine aspartate and glutamate metabolism (hsa00250), Complement and coagulation cascades (hsa04610), Protein processing in the endoplasmic reticulum (hsa04141), etc.

### 3.4 Multiphase drug repurposing strategy based on identified biomarkers

The integrated approach of drug repurposing, which utilized connectivity map analysis and drug-gene interaction text-mining, etc, facilitated the creation of a library of potential repurposed drugs. This resulted in the identification of 81 potential candidate drugs. All these candidates have been utilized for molecular docking to analyze further interactions (details of the drugs are given in Supplementary Material S4). Among these 81 drugs, DGIdb (The Drug Gene Interaction Database) based screening identified a total of 19 drugs. The drugs with the highest interaction scores (IS) were Vigabatrin (IS- 11.79601078), Divalproex sodium (IS- 8.847008084), and Pyruvic Acid (IS-5.89800539). Screening using the Drug Repurposing Encyclopedia (DRE) database identified a total of 55 drugs, with Scopolamine (Enrichment score (ES) - 0.973), Amiloride (ES - 0.971), Damnacanthal (ES - 0.967), and Esculin (ES - 0.962) showing the highest enrichment scores. In addition, the search conducted by the Command app revealed a total of 6 drugs, including Glibenclamide, Phenelzine, etc.

### 3.5 Drug-target interaction analysis through molecular docking and MM-GBSA calculations

**FIGURE 7:**
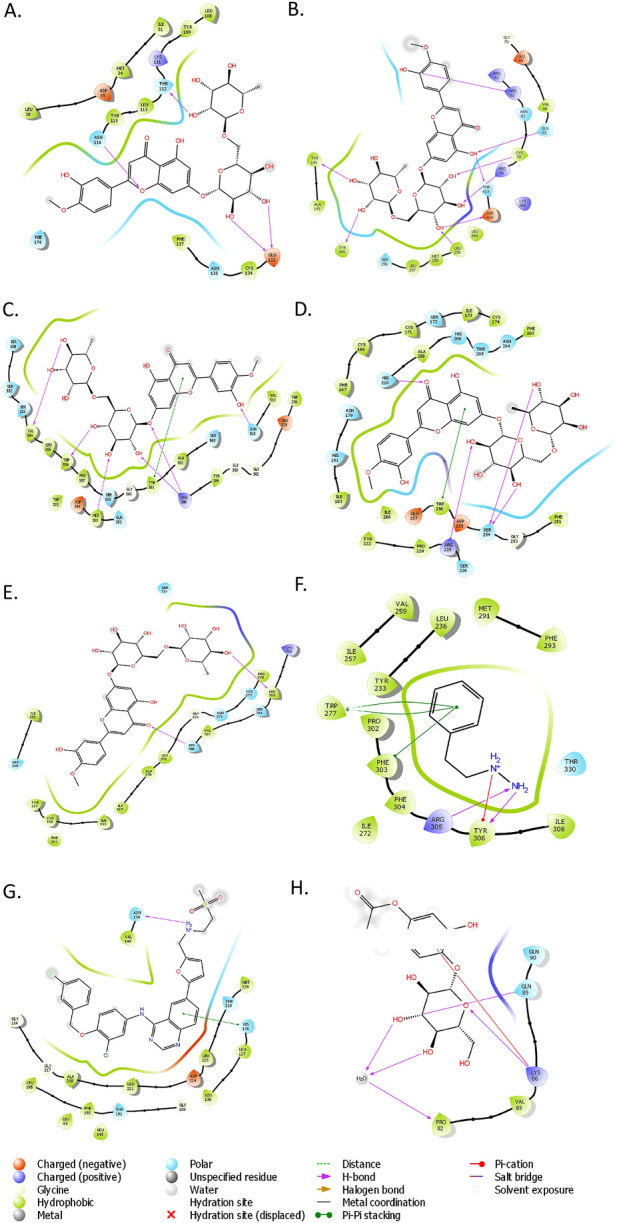
**(A)** ZFP1 with Diosmin, **(B)** C8B with Diosmin, **(C)** MBTPS1 with Diosmin, **(D)** CENPV with Diosmin, **(E)** ABCB11 with Diosmin, **(F)** FBXL3 with Phenelzine, **(G)** APOF with Lapatinib, and **(H)** ABAT with Esculin.

Docking results showed good interaction between ZFP1, C8B, MBPTS1, CENPV, ABCB11, and Diosmin with docking scores of −11.6821 kcal/mol, −11.134 kcal/mol, 10.4712 kcal/mol, −10.2391 kcal/mol, and −9.85606 kcal/mol respectively (Figures 7A–E). There are three hydrogen bonds between ZFP1 and Diosmin at THR112, ASN116 and GLU133 positions (Figure 7A) and eight hydrogen bonds between C8B and Diosmin at positions–ARG82, GLN65, CYS79, THR423, ASP424, LEU259, TYR166 and TYR141 (Figure 7B). The hydrogen bond interactions between MBPTS1 and Diosmin found at VAL54, TRP556, MET353, ARG386, and ASN515 (Figure 7C) and the interactions between CENPV and Diosmin included Hydrogen bonds at HIS210, ARG225, SER254 positions and PI-PI interactions at TYR561 and TRP256 positions (Figure 7D). The details about the interactions between top protein-ligand complexes are provided in Supplementary Material S5. ABAT and Esculin with a docking score of −4.30418 kcal/mol (Figure 7H). APOF and Lapatinib with a docking score of −6.90333 kcal/mol (Figure 7G), indicating a strong interaction between the protein and ligand. A satisfactory interaction has also been observed between FBX23 and Phenelzine with a docking score of −6.30952 kcal/mol (Figure 7F). The MM-GBSA analysis supported the docking results, showing strong binding affinities. CENPV-Diosmin had a binding free energy of −100.71 kcal/mol, with Van der Waals and electrostatic contributions of −49.50 and −46.93 kcal/mol, respectively. Similarly, C8B-Diosmin exhibited a binding free energy of −74.58 kcal/mol, with Van der Waals and electrostatic contributions of −37.40 and −52.24 kcal/mol (The detailed result of MM-GBSA analysis of the top ligand-protein complexes were provided as Supplementary Material S6). These results highlight a significant binding affinity driven by a balanced interplay of electrostatic, van der Waals, and lipophilic interactions.

## 4 Discussion

Identification of biomarkers associated with NAFLD-driven HCC through conventional genomics-based methods is not an easy task, as the disease progresses through several stages over a prolonged period of time (Xu et al., 2023). The present work highlights the implementation of machine learning methods over conventional strategies in prognostic biomarker identification. The selection of an appropriate classifier for disease stage classification was the first step of this study, as different data types respond differently to various classifiers (de Amorim et al., 2023). It is essential to choose a classifier that is well-suited to the specific characteristics of the data to achieve optimal performance. Factors such as the distribution of the data, the presence of noise, and the complexity of the relationships between features can all influence the effectiveness of a given classifier (Saseendran et al., 2019). DISCR emerged as the most appropriate classifier in this analysis, exhibiting superior performance and reliability compared to other classifiers. The advantage of DISCR is that it can model intricate decision boundaries by discriminating between classes in a diverse set of data (Meyer-Baese and Schmid, 2014). Consequently, it was chosen for the subsequent steps of this study. Given that the primary objective of this work is to identify the key genes involved in the stage-wise progression of NAFLD to HCC, the main focus was on finding genes that are consistently expressed across all stages of the disease. While mutual information-based feature selection methods (such as JMI, MIM, and NMIFS) can effectively capture dependencies between variables, they also have potential disadvantages, such as high computational complexity and sensitivity to noise. To overcome these limitations, a variety of alternative feature selection methods were employed, including filter-based methods like ReliefF, Cramér’s V, Kendall’s Tau, Pearson correlation, and Spearman correlation coefficient, as well as embedded methods like LASSO, Ridge regression, and gradient boosting techniques. These methods help in addressing different aspects of feature selection. For example, filter-based methods assess the individual importance of each feature, without considering how they relate to each other (Bellotti et al., 2014), while embedded methods integrate feature selection into the model training process, simultaneously selecting the most relevant features, enabling more flexible and refined selection by considering how features interact with each other and influence the target variable (Bouchlaghem et al., 2022). This ensemble method enhances feature importance, improving model performance and identifying a robust set of ten co-expressed genes linked to disease progression. These genes include *C8B, APOF, FBXL3, ABAT, ZFP1, MBTPS1, CENPV, METTL23, RPL9*, and *ABCB11*. The pathway enrichment analysis results suggest that these genes are primarily involved in various pathways, including metabolism-related pathways such as Alanine, Aspartate, and Glutamate Metabolism, Propanoate Metabolism, Butanoate Metabolism, and Valine, Leucine, and Isoleucine Degradation. They are also involved in protein processing pathways like protein processing in the Endoplasmic Reticulum and Ribosome, as well as inflammation and immunological pathways like Complement and Coagulation Cascades and Systemic Lupus Erythematosus. These results indicate the involvement of both protein and lipid metabolism and inflammation in disease progression. Metabolic events specifically protein and lipid metabolism as well as ER-Mitochondrial dysregulation due to high metabolic stress is a long-suspected event for NAFLD to HCC progression (Zheng et al., 2023; Léveillé and Estall, 2019). Metabolic dysregulation in this disease progression is likely linked to inflammation and oxidative damage, which contribute to the onset of cirrhosis and the eventual development of liver cancer. This significant involvement of metabolism-related pathways also suggests that this evaluated energy production is possibly required for the rapid growth and division of cancer cells, a primary feature of cancer cells (Phan et al., 2014). Furthermore, out of the ten identified genes, eight genes *ABAT, C8B, FBXL3, ZFP1, ABCB11, MBTPS1, CENPV,* and *APOF* showed a strong association with lower overall survival rates in patients with HCC. The high hazard ratios (HR) for these genes indicate that patients with higher expression levels of these genes tend to have a poorer prognosis, such as a shorter overall survival time. The *ABAT* gene encodes the 4-Aminobutyrate Aminotransferase which is crucial for the catabolism of inhibitory neurotransmitters like GABA-transaminase (Besse et al., 2015). Altered expression of this gene has been observed in breast cancer (Chen et al., 2019), and its involvement in tumorigenesis and tumor immunity in HCC is a recent finding (Gao et al., 2022). The *C8B* gene encodes the beta subunit of complement complex 8 (Zhang Y. et al., 2021), which has recently been found to have predictive potential in hepatocellular carcinoma (HCC) development (Xiao et al., 2022). *FBXL3* encodes for an F-box and leucine-rich repeat protein 3, which plays a vital role in regulating circadian rhythm (Fagiani et al., 2022). It works together with *CRY2* to degrade the C-MYC protein, which helps prevent tumor growth (Huber et al., 2016b). *FBXL3* has been previously reported as an important cancer marker (Huber et al., 2016a). The *ZFP1* gene encodes zinc finger motif proteins, which play a crucial role in several transcriptional activation and repression processes (Li X. et al., 2022). *ABCB11* encodes the primary ABC transporter, which is called the bile salt export pump (BSEP), in hepatic cells (Sohail et al., 2021). Malfunctioning BSEP is particularly significant in liver malignancies (Lagana et al., 2015). *MBTPS1*, which encodes the Membrane-Bound Transcription Factor Peptidase protein, has been implicated in the process of cancer cell proliferation (Hartal-Benishay et al., 2022). *CENPV* encodes Centromere Protein V, a vital component involved in the process of mitosis and exhibiting significant upregulation in several cancer types (Zhang S. et al., 2021). The *APOF* gene encodes Apolipoprotein F, which plays a role in lipid metabolism by binding to LDL and VLDL (Deprince et al., 2023). While its exact mechanism in HCC is not completely understood, it has been shown to act as a tumor suppressor and could be a promising target for therapeutic development in HCC. Subsequently, as these genes were identified as key modulators involved in this progression, they were further screened as targets to identify potential drug candidates utilizing DGIdb, Drug Repurposing Encyclopedia, and the COMMAND app. These databases utilize several data sources including gene expression data and approaches, such as expert curation, text-mining, etc to discover possible therapeutic interventions. The resulting combined list of 81 potential drugs was further screened through molecular docking and MM-GBSA analysis. The analysis revealed a strong interaction between the drug Diosmin and targets such as ZFP1, C8B, MBPTS1, CENPV, and ABCB11, characterized by numerous hydrogen bonds and pi-pi interactions. Diosmin is reported to have anti-inflammatory, antioxidative, insulin-sensitizing, antimutagenic, and antiulcer properties, and is widely being used for the improvement of blood-related insufficiencies (Huwait and Mobashir, 2022). ABAT had a robust interaction with Esculin, whilst APOF and FBX23 demonstrated notable interactions with Lapatinib and Phenelzine, respectively. Esculin is known to have anti-inflammatory properties and is used in multiple disorders like arthritis, ulcerative colitis, etc (Cai and Cai, 2023). Whereas, Lapatinib is reportedly used in breast cancer treatment (Opdam et al., 2012) and Phenelzine is a widely used drug in panic disorders, Chronic resistant depression, etc (Blanco et al., 2010). Since there are no direct medications available for idiopathic HCC, including those induced by NAFLD, and given that the pathogenesis of non-idiopathic HCC differs from idiopathic HCC, drug repurposing to target these mechanisms can significantly reduce the development time and cost of new treatments. This also ensures better patient outcomes using already approved drugs with known safety profiles. These findings indicate promising therapeutic paths that should be further investigated in clinical settings by experts.

## 5 Conclusion

In conclusion, ensemble feature selection framework used in this study improves the discrimination and stability of the final selected features. Using an ensemble feature selection approach, this study successfully identified key biomarkers, including *ABAT, C8B, FBXL3,* and *ZFP1*, providing valuable insights into NAFLD to HCC disease progression. A drug repurposing approach identified therapeutic agents, including Diosmin, Esculin, and Lapatinib, that were found to be effective against these marker genes. These findings offer a strong foundation for future research and therapeutic development in the treatment of NAFLD-mediated HCC. The integration of biomarker prediction with drug repurposing could enhance precision medicine approaches, paving the way for more effective and targeted treatments.

## Data Availability

The original contributions presented in the study are included in the article/Supplementary Material, further inquiries can be directed to the corresponding authors.

## Glossary

NAFLD: Non-Alcoholic Fatty Liver Disease
NASH: Non-Alcoholic Steatohepatitis
HCC: Hepatocellular Carcinoma
ML: Machine Learning
SVM: Support Vector Machine
ANN: Artificial Neural Network
KNN: k-Nearest Neighbors
DISCR: Discriminant Analysis
RF: Random Forest
DT: Decision Tree
NB: Naive Bayes
CIFE: Conditional Informative Feature Extraction
JMI: Joint Mutual Information
MIM: Mutual Information Maximization/Maximum Relevance
LASSO: Least Absolute Shrinkage and Selection Operator
GO: Gene Ontology
KEGG: Kyoto Encyclopedia of Genes and Genomes
ER: Endoplasmic Reticulum
GEO: Gene Expression Omnibus
NCBI: National Center for Biotechnology Information
RMA: Robust Multichip Averaging
RMR: Relevant Mutual Redundancy
DISR: Double Input Symmetrical Relevance
NMIFS: Normalized Mutual Information Feature Selection
DAVID: Database for Annotation, Visualization, and Integrated Discovery
FDR: False Discovery Rate
TCGA-LIHC: The Cancer Genome AtlasLiver Hepatocellular Carcinoma
HR: Hazard Ratio
CI: Confidence Interval
DGIdb: The Drug Gene Interaction Database
MSigDB: Molecular Signatures Database
DREIMT: Drug Response Element Interactions Modelling Tool
CMap: Connectivity Map
PDB: Protein Data Bank
RCSB: Research Collaboratory for Structural Bioinformatics
BLAST: Basic Local Alignment Search Tool
OPLS: Optimized Potentials for Liquid Simulations
