# Supplementary material for: Biomarker-driven drug repurposing for NAFLD-associated hepatocellular carcinoma using machine learning integrated ensemble feature selection
Source: Front Bioinform. 2025 Apr 17;5:1522401. doi: 10.3389/fbinf.2025.1522401 (PMC12043677; doi:10.3389/fbinf.2025.1522401)
Supplement: Supplementary file 1 [file Table1.docx]

Supplementary file 1: Information on the sample sizes and characteristics.

| Accession ID | Organism | Array | Sample type | Sample IDs | Contributors | Reference |
| --- | --- | --- | --- | --- | --- | --- |
| GSE48452 | *Homo sapiens* | [HuGene-1_1-st] Affymetrix Human Gene 1.1 ST Array | Healthy | GSM1178970  GSM1178971  GSM1178972  GSM1178973  GSM1178974  GSM1178977  GSM1178978  GSM1178979  GSM1178998  GSM1179009  GSM1179010  GSM1179018  GSM1179024  GSM1179031 | Jochen H, Andreas T, Timo I | PMID:23931760 |
|  |  |  | Obese | GSM1178976  GSM1178981  GSM1178982  GSM1178983  GSM1178984  GSM1178985  GSM1178990  GSM1178991  GSM1178992  GSM1178994  GSM1178997  GSM1179000  GSM1179005  GSM1179007  GSM1179012  GSM1179013  GSM1179014  GSM1179016  GSM1179019  GSM1179020  GSM1179022  GSM1179028  GSM1179030  GSM1179032  GSM1179038  GSM1179041  GSM1179042 |  |  |
|  |  |  | NAFLD(Steatosis) | GSM1178986  GSM1178988  GSM1178989  GSM1178993  GSM1178999  GSM1179011  GSM1179021  GSM1179023  GSM1179025  GSM1179027  GSM1179029  GSM1179034  GSM1179037  GSM1179040 |  |  |
|  |  |  | NASH | GSM1178975  GSM1178980  GSM1178987  GSM1178995  GSM1178996  GSM1179001  GSM1179002  GSM1179003  GSM1179004  GSM1179006  GSM1179008  GSM1179015  GSM1179017  GSM1179026  GSM1179033  GSM1179035  GSM1179036  GSM1179039 |  |  |
| GSE25097 | *Homo sapiens* | Rosetta/Merck Human RSTA Affymetrix 1.0 microarray, Custom CDF | HCC | GSM617304  GSM617305  GSM617306  GSM617307  GSM617308  GSM617309  GSM617310  GSM617311  GSM617312  GSM617313  GSM617314  GSM617315  GSM617316  GSM617317  GSM617318  GSM617319  GSM617320  GSM617321  GSM617322  GSM617323  GSM617324  GSM617325  GSM617326  GSM617327  GSM617328  GSM617329  GSM617330  GSM617331  GSM617332  GSM617333  GSM617334  GSM617335  GSM617336  GSM617337  GSM617338  GSM617339  GSM617340  GSM617341  GSM617342  GSM617343  GSM617344  GSM617345  GSM617346  GSM617347  GSM617348  GSM617349  GSM617350  GSM617351  GSM617352  GSM617353  GSM617354  GSM617355  GSM617356  GSM617357  GSM617358  GSM617359  GSM617360  GSM617361  GSM617362 | Zhang C | PMID: 21949730 |
