# Supplementary figures and images for: Biomarker-driven drug repurposing for NAFLD-associated hepatocellular carcinoma using machine learning integrated ensemble feature selection
Source: Front Bioinform. 2025 Apr 17;5:1522401. doi: 10.3389/fbinf.2025.1522401 (PMC12043677; doi:10.3389/fbinf.2025.1522401)

Supplementary file 2: Performance metrics for different feature selection models (E-L).


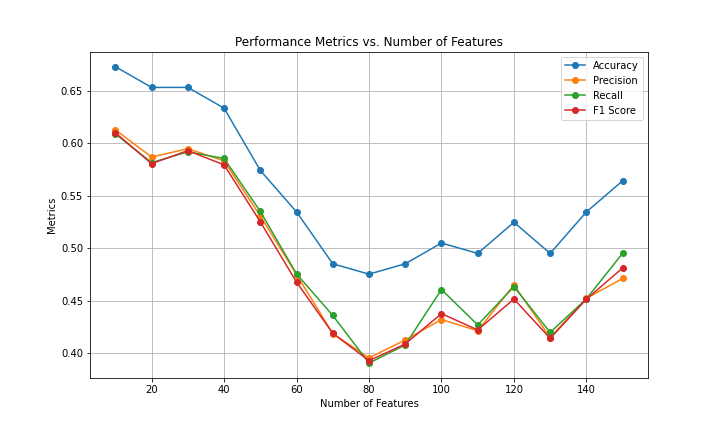

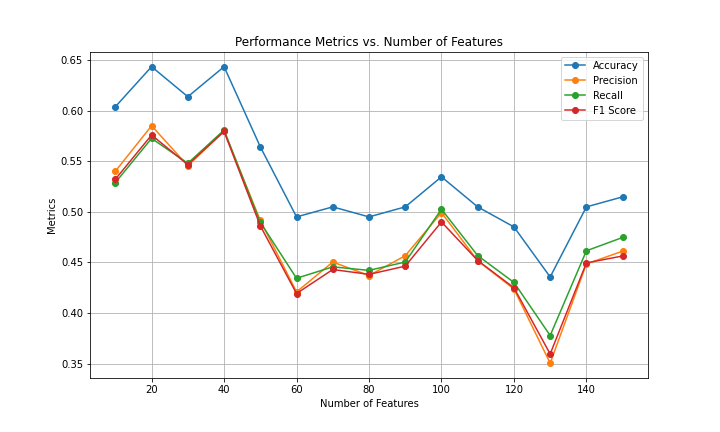

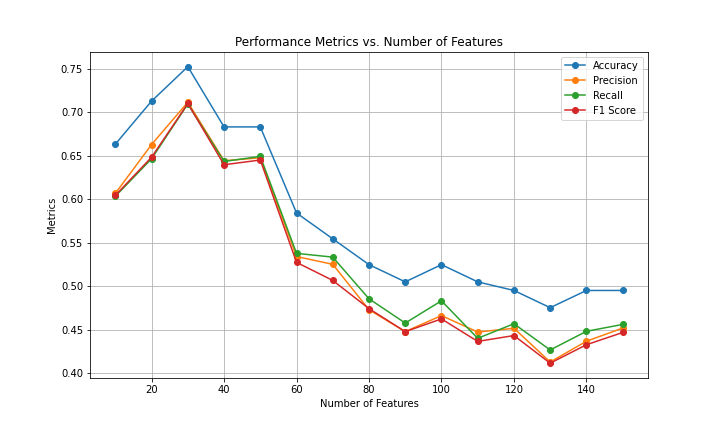

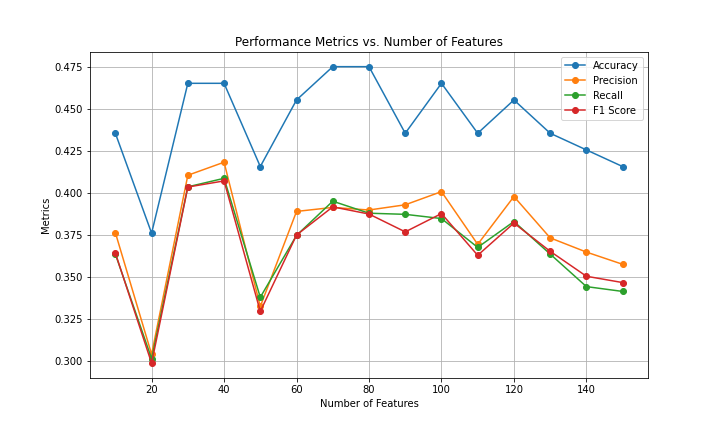

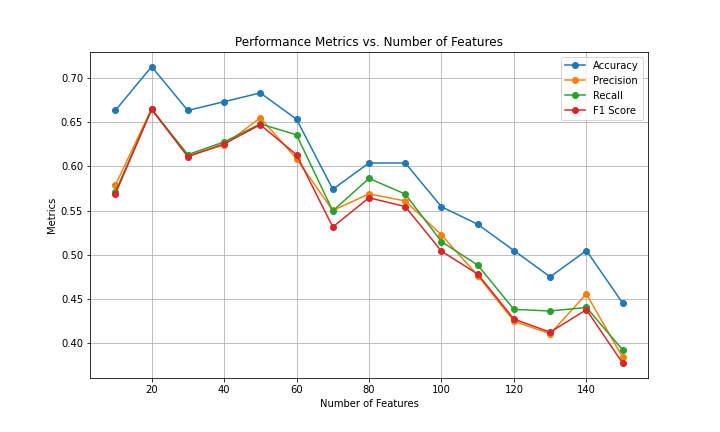

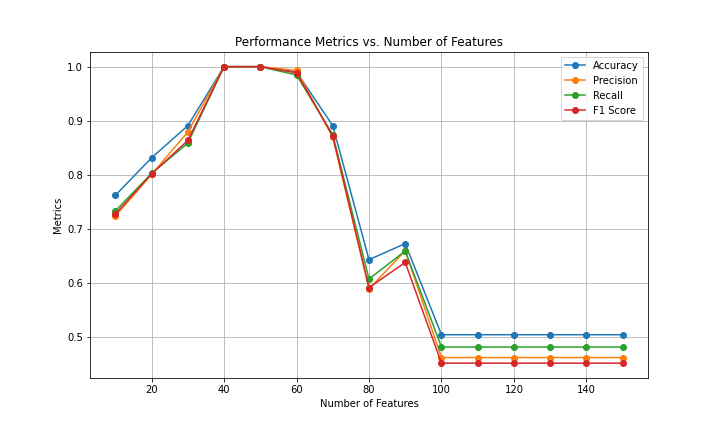

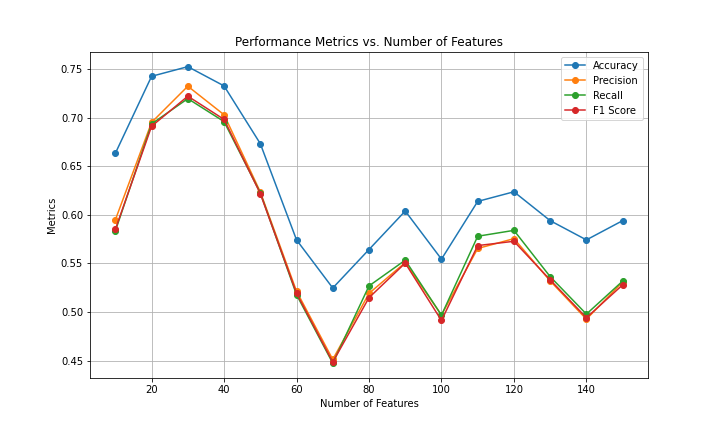

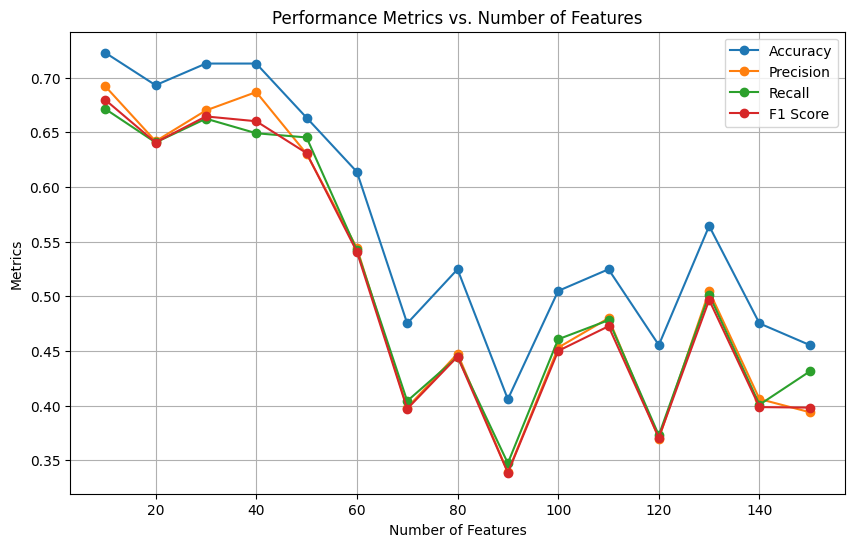


E.

F.

G.

H.

I.

J.

K.

L.

Supplement: Supplementary file 2 [file Table2.docx]
