# Supplementary material for: Biomarker-driven drug repurposing for NAFLD-associated hepatocellular carcinoma using machine learning integrated ensemble feature selection
Source: Front Bioinform. 2025 Apr 17;5:1522401. doi: 10.3389/fbinf.2025.1522401 (PMC12043677; doi:10.3389/fbinf.2025.1522401)
Supplement: Supplementary file 3 [file Table5.docx]

**Supplementary file 5: Molecular docking analysis results of the best-docked complexes.**

| **Protein** | **Compounds** | **PubChem ID** | **Binding energy** | **H-bonds** | **π-π interactions** |
| --- | --- | --- | --- | --- | --- |
| C8B | Diosmin | 5281613 | -11.134 | ARG82,  GLN65,  CYS79,  ARG78,  THR423,  ASP424,  LEU259,  TYR160,  TYR141 | - |
| ABCB11 | Diosmin | 5281613 | -9.856 | HIS708,  LEU703 | - |
| APOF | Lapatinib | 208908 | -6.903 | ASN139 | HIS126 |
| ABAT | Esculin | 5281417 | -4.304 | GLN89,  LYS86,  PRO82 | - |
| CENPV | Diosmin | 5281613 | -10.239 | HIS210,  ARG225,  SER254 | TRP256 |
| FBXL3 | Phenelzine | 3675 | -6.3095 | ARG305,  TYR306 | TRP277,  PHE303 |
| MBTPS1 | Diosmin | 5281613 | -10.471 | VAL554,  TRP556,  MET353,  ARG386,  ASN515 | TYR561 |
| ZFP1 |  | 5281613 | -11.682 | THR112,  ASN116,  GLU133 | - |
