# Supplementary material for: Biomarker-driven drug repurposing for NAFLD-associated hepatocellular carcinoma using machine learning integrated ensemble feature selection
Source: Front Bioinform. 2025 Apr 17;5:1522401. doi: 10.3389/fbinf.2025.1522401 (PMC12043677; doi:10.3389/fbinf.2025.1522401)
Supplement: Supplementary file 4 [file Table3.docx]

Supplementary file 3: Survival curve analysis results of identified genes through ensemble feature selection(E-J).

E.


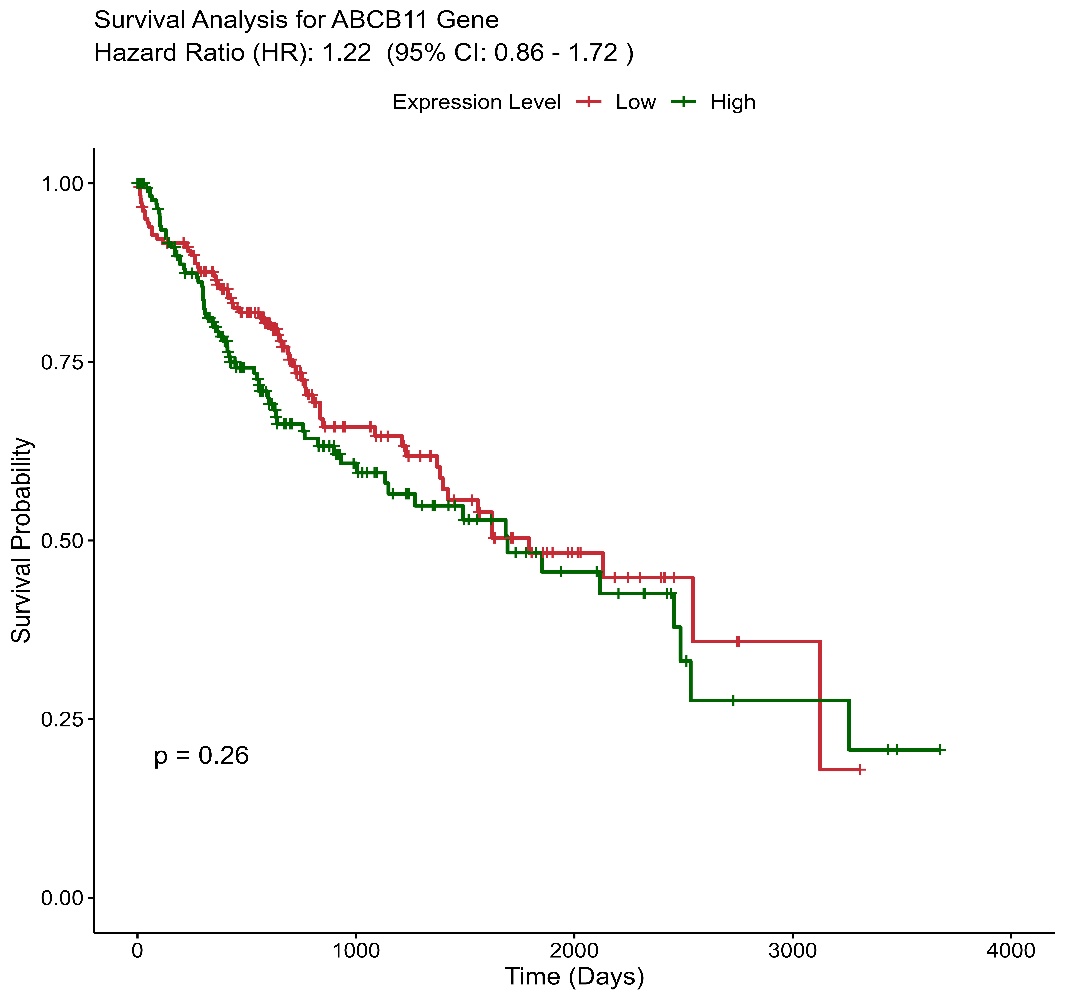

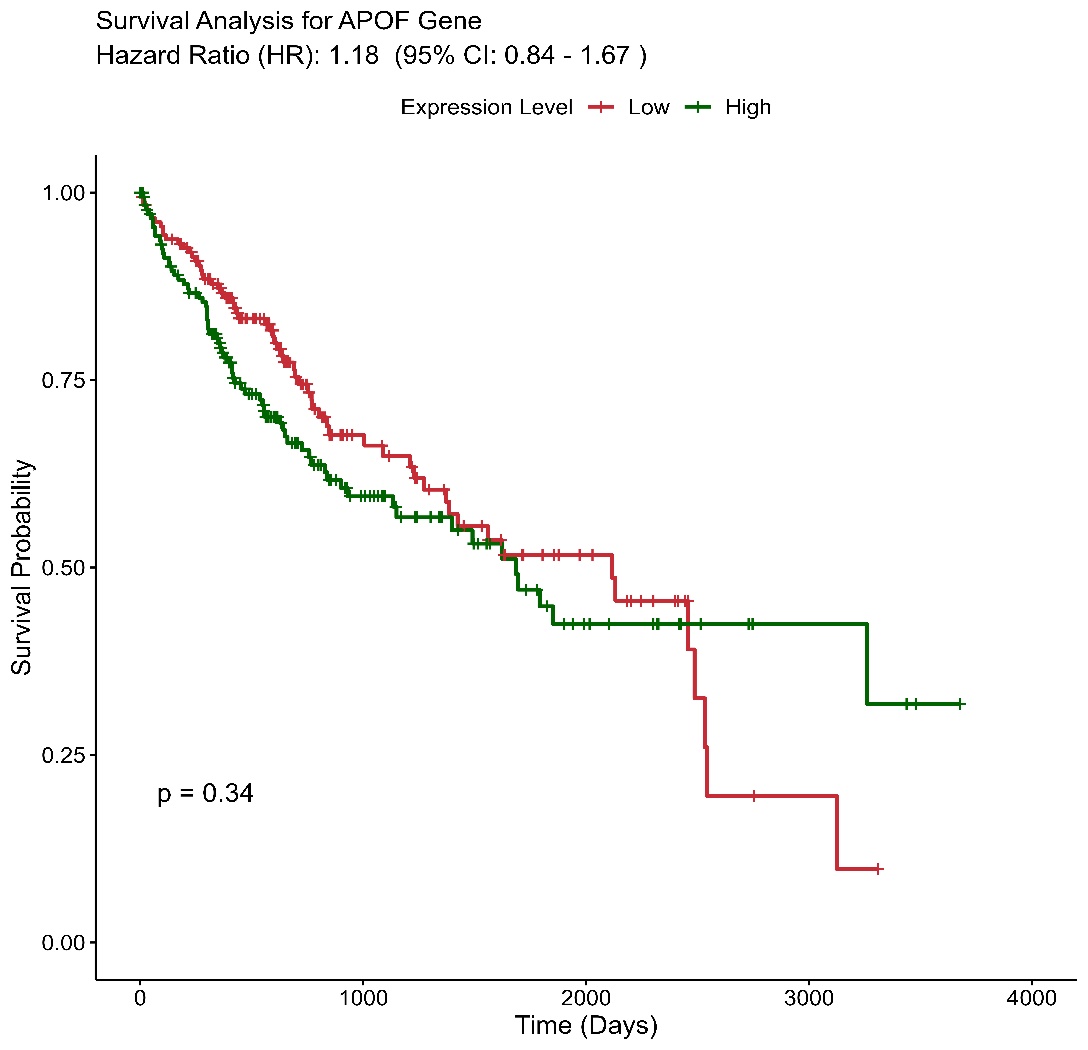

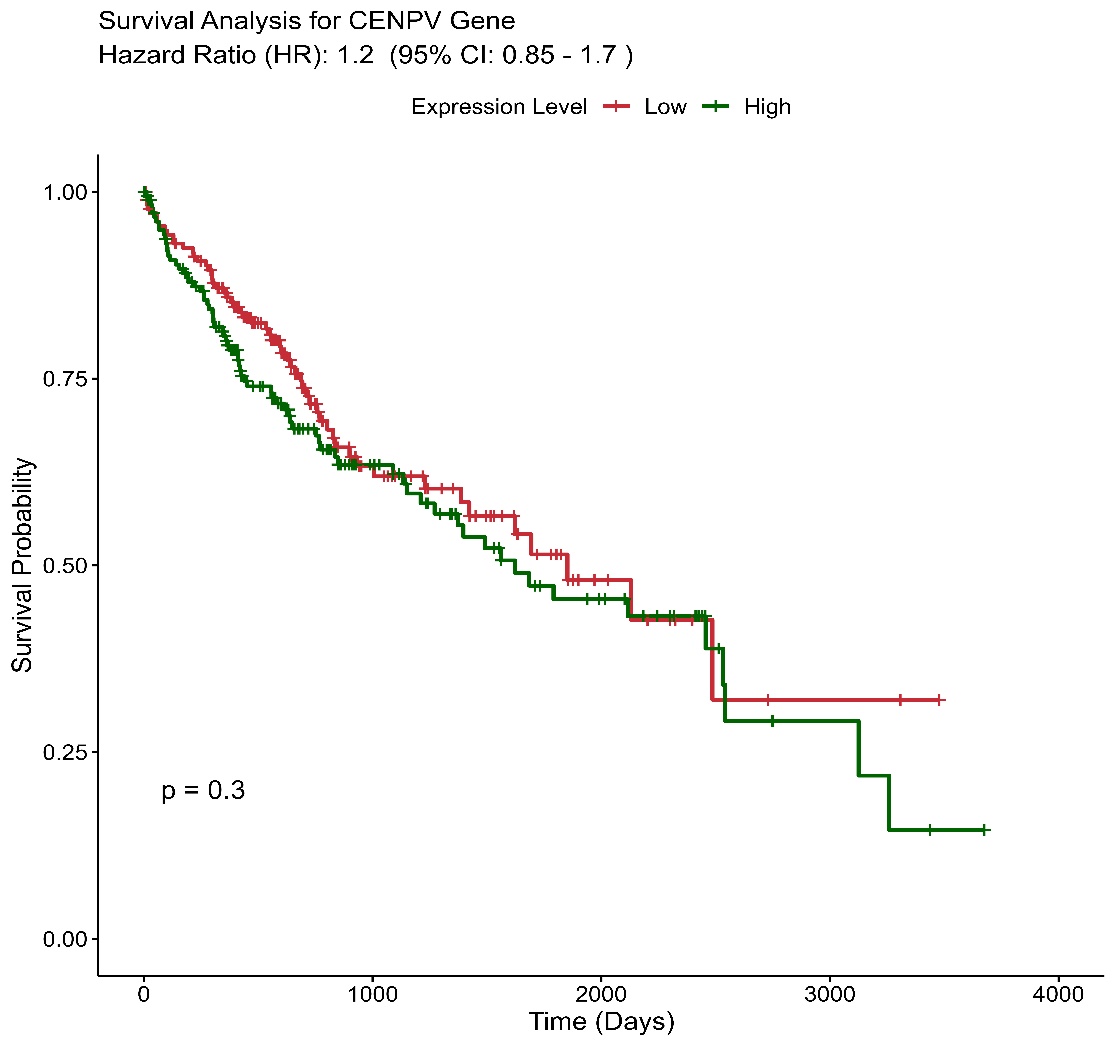

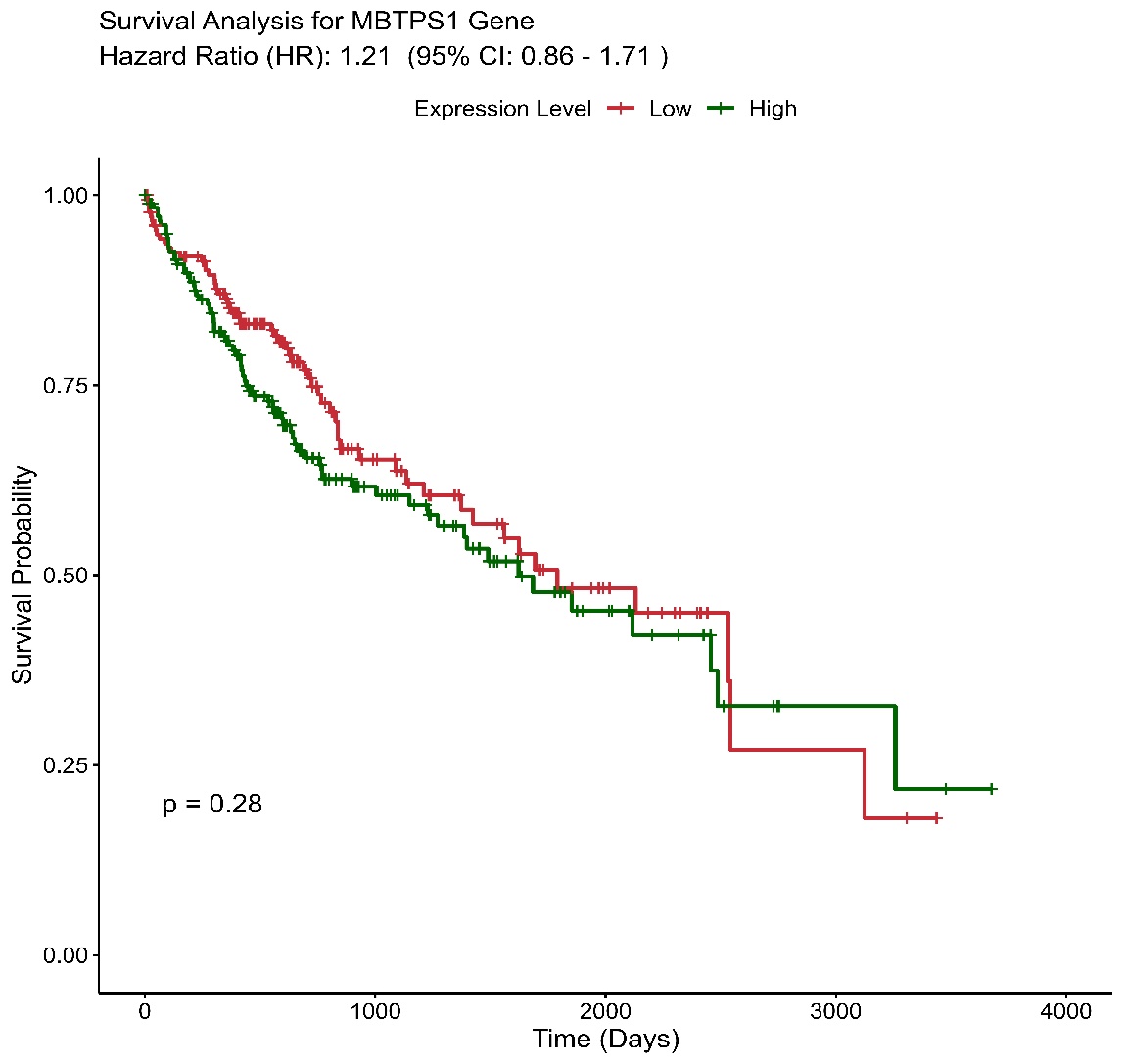

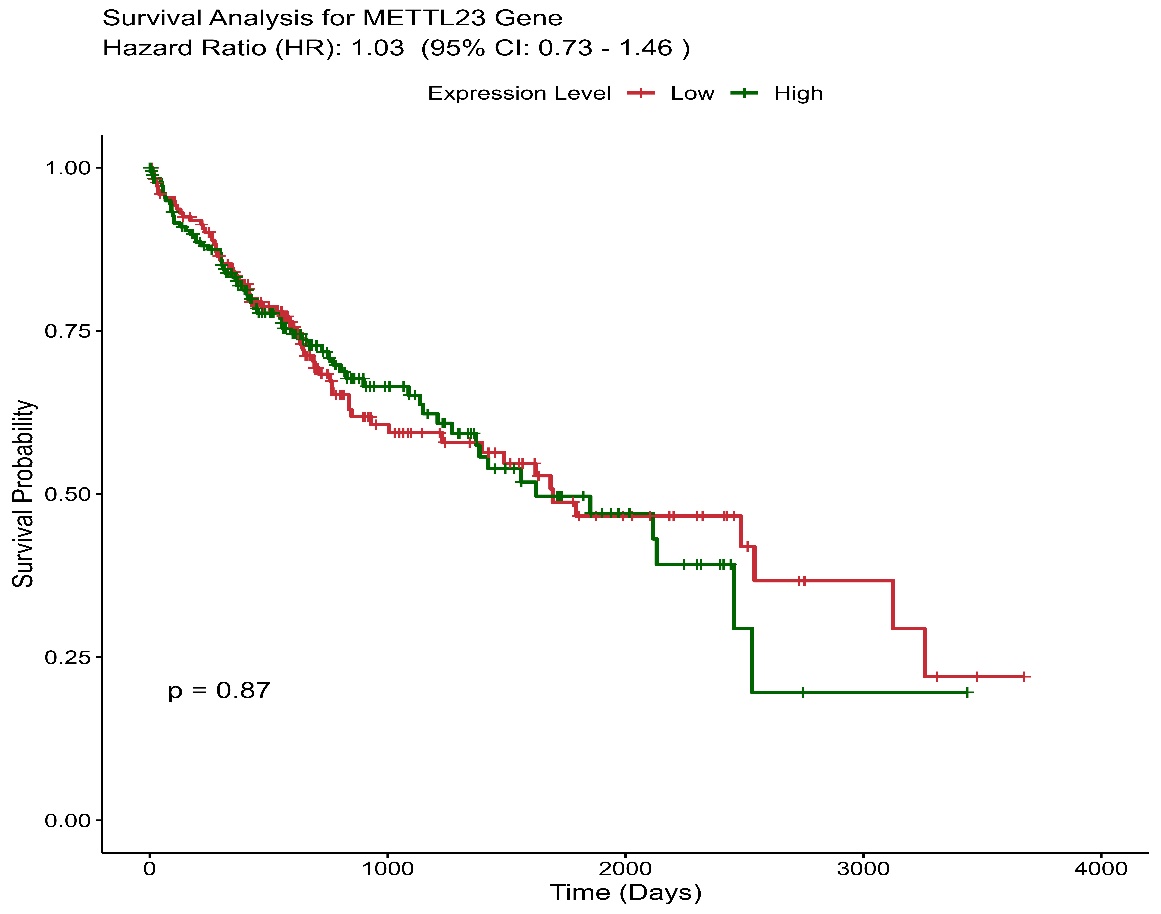

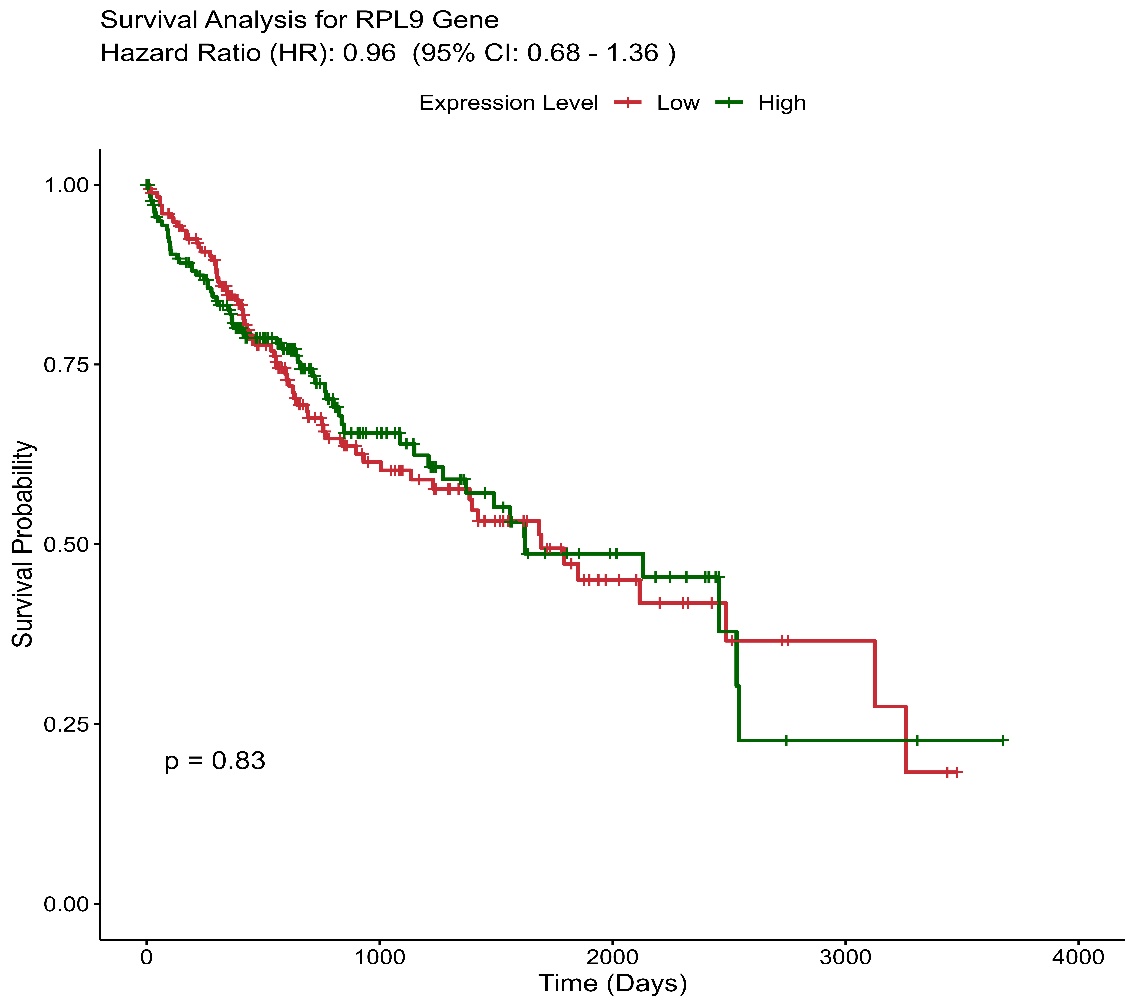


F.

G.

H.

I.

J.
