# Supplementary material for: Biomarker-driven drug repurposing for NAFLD-associated hepatocellular carcinoma using machine learning integrated ensemble feature selection
Source: Front Bioinform. 2025 Apr 17;5:1522401. doi: 10.3389/fbinf.2025.1522401 (PMC12043677; doi:10.3389/fbinf.2025.1522401)
Supplement: Supplementary file 5 [file Table4.docx]

Supplementary file 4: Total list of Drugs and their respective PubChem IDs, identified through Drug Repurposing.

| **Drug Names** | **PubChem IDs** |
| --- | --- |
| cholic acid | 221493 |
| Su-6656 | [5312137](https://pubchem.ncbi.nlm.nih.gov/compound/5312137) |
| Psb-36 | [11689583](https://pubchem.ncbi.nlm.nih.gov/compound/11689583) |
| Pyridine-2-Aldoxime | [135423666](https://pubchem.ncbi.nlm.nih.gov/compound/135423666) |
| Amiloride | [16231](https://pubchem.ncbi.nlm.nih.gov/compound/16231) |
| Ac-55649 | [1714884](https://pubchem.ncbi.nlm.nih.gov/compound/1714884) |
| Dpo-1 | [21678144](https://pubchem.ncbi.nlm.nih.gov/compound/21678144) |
| Clofazimine | [2794](https://pubchem.ncbi.nlm.nih.gov/compound/2794) |
| glibenclamide | [3488](https://pubchem.ncbi.nlm.nih.gov/compound/3488) |
| Dicyclohexylurea | [4277](https://pubchem.ncbi.nlm.nih.gov/compound/4277) |
| Naftopidil | [4418](https://pubchem.ncbi.nlm.nih.gov/compound/4418) |
| probucol | [4912](https://pubchem.ncbi.nlm.nih.gov/compound/4912) |
| cyclosporin-a | [5284373](https://pubchem.ncbi.nlm.nih.gov/compound/5284373) |
| Etiocholanolone | [5880](https://pubchem.ncbi.nlm.nih.gov/compound/5880) |
| chenodeoxycholic acid | [10133](https://pubchem.ncbi.nlm.nih.gov/compound/10133) |
| Epitestosterone | [10204](https://pubchem.ncbi.nlm.nih.gov/compound/10204) |
| pyridoxal phosphate anhydrous | [1051](https://pubchem.ncbi.nlm.nih.gov/compound/1051) |
| pyruvic acid | [1060](https://pubchem.ncbi.nlm.nih.gov/compound/1060) |
| Rx-821002 | [108094](https://pubchem.ncbi.nlm.nih.gov/compound/108094) |
| Way-213613 | [11531745](https://pubchem.ncbi.nlm.nih.gov/compound/11531745) |
| Scopolamine | [11968014](https://pubchem.ncbi.nlm.nih.gov/compound/11968014) |
| Saclofen | [122150](https://pubchem.ncbi.nlm.nih.gov/compound/122150) |
| Trimetozine | [12478](https://pubchem.ncbi.nlm.nih.gov/compound/12478) |
| 4-hydroxybenzaldehyde | [126](https://pubchem.ncbi.nlm.nih.gov/compound/126) |
| Amthamine | [126688](https://pubchem.ncbi.nlm.nih.gov/compound/126688) |
| Cyanopindolol | [155346](https://pubchem.ncbi.nlm.nih.gov/compound/155346) |
| Zd-7288 | [162640412](https://pubchem.ncbi.nlm.nih.gov/compound/162640412) |
| pravastatin sodium | [16759173](https://pubchem.ncbi.nlm.nih.gov/compound/16759173) |
| hydroxyprogesterone caproate (USP) | [169870](https://pubchem.ncbi.nlm.nih.gov/compound/169870) |
| lapatinib | [208908](https://pubchem.ncbi.nlm.nih.gov/compound/208908) |
| Anabasine | [2181](https://pubchem.ncbi.nlm.nih.gov/compound/2181) |
| Bepridil | [2351](https://pubchem.ncbi.nlm.nih.gov/compound/2351) |
| divalproex sodium | [23663956](https://pubchem.ncbi.nlm.nih.gov/compound/23663956) |
| GS-9256 | [24823649](https://pubchem.ncbi.nlm.nih.gov/compound/24823649) |
| Carmofur | [2577](https://pubchem.ncbi.nlm.nih.gov/compound/2577) |
| Chlorpromazine | [2726](https://pubchem.ncbi.nlm.nih.gov/compound/2726) |
| Damnacanthal | [2948](https://pubchem.ncbi.nlm.nih.gov/compound/2948) |
| valproic acid | [3121](https://pubchem.ncbi.nlm.nih.gov/compound/3121) |
| valproic-acid | [3121](https://pubchem.ncbi.nlm.nih.gov/compound/3121) |
| Domperidone | [3151](https://pubchem.ncbi.nlm.nih.gov/compound/3151) |
| Ethaverine | [3280](https://pubchem.ncbi.nlm.nih.gov/compound/3280) |
| Fluconazole | [3365](https://pubchem.ncbi.nlm.nih.gov/compound/3365) |
| glyburide | [3488](https://pubchem.ncbi.nlm.nih.gov/compound/3488) |
| Hexetidine | [3607](https://pubchem.ncbi.nlm.nih.gov/compound/3607) |
| phenelzine | [3675](https://pubchem.ncbi.nlm.nih.gov/compound/3675) |
| Vecuronium | [39765](https://pubchem.ncbi.nlm.nih.gov/compound/39765) |
| Nornicotine | [412](https://pubchem.ncbi.nlm.nih.gov/compound/412) |
| Linezolid | [441401](https://pubchem.ncbi.nlm.nih.gov/compound/441401) |
| rosuvastatin | [446157](https://pubchem.ncbi.nlm.nih.gov/compound/446157) |
| Ku-C104233 | [44623839](https://pubchem.ncbi.nlm.nih.gov/compound/44623839) |
| Procaterol | [4916](https://pubchem.ncbi.nlm.nih.gov/compound/4916) |
| Quipazine | [5011](https://pubchem.ncbi.nlm.nih.gov/compound/5011) |
| Ex-527 | [5113032](https://pubchem.ncbi.nlm.nih.gov/compound/5113032) |
| Esculin | [5281417](https://pubchem.ncbi.nlm.nih.gov/compound/5281417) |
| Diosmin | [5281613](https://pubchem.ncbi.nlm.nih.gov/compound/5281613) |
| cyclosporine | [5284373](https://pubchem.ncbi.nlm.nih.gov/compound/5284373) |
| Fluticasone | [5311101](https://pubchem.ncbi.nlm.nih.gov/compound/5311101) |
| stiripentol | [5311454](https://pubchem.ncbi.nlm.nih.gov/compound/5311454) |
| Idazoxan | [54459](https://pubchem.ncbi.nlm.nih.gov/compound/54459) |
| Scandenin | [54676535](https://pubchem.ncbi.nlm.nih.gov/compound/54676535) |
| Warfarin | [54678486](https://pubchem.ncbi.nlm.nih.gov/compound/54678486) |
| Demeclocycline | [54680690](https://pubchem.ncbi.nlm.nih.gov/compound/54680690) |
| Chlortetracycline | [54680690](https://pubchem.ncbi.nlm.nih.gov/compound/54680690) |
| Dihydrorobustic-Acid | [54686939](https://pubchem.ncbi.nlm.nih.gov/compound/54686939) |
| vigabatrin | [5665](https://pubchem.ncbi.nlm.nih.gov/compound/5665) |
| alanine | [5950](https://pubchem.ncbi.nlm.nih.gov/compound/5950) |
| Zolmitriptan | [60857](https://pubchem.ncbi.nlm.nih.gov/compound/60857) |
| Bh3i-1 | [6090108](https://pubchem.ncbi.nlm.nih.gov/compound/6090108) |
| Penicillin | [6098](https://pubchem.ncbi.nlm.nih.gov/compound/6098) |
| Cephalotaxine | [65305](https://pubchem.ncbi.nlm.nih.gov/compound/65305) |
| Cyclizine | [6726](https://pubchem.ncbi.nlm.nih.gov/compound/6726) |
| Toltrazuril | [68591](https://pubchem.ncbi.nlm.nih.gov/compound/68591) |
| Dantrolene | [6914273](https://pubchem.ncbi.nlm.nih.gov/compound/6914273) |
| DP-VPA | [6918692](https://pubchem.ncbi.nlm.nih.gov/compound/6918692) |
| Dihydroobliquin | 785 |
| Peucedanin | [8616](https://pubchem.ncbi.nlm.nih.gov/compound/8616) |
| tiagabine hydrochloride | [91274](https://pubchem.ncbi.nlm.nih.gov/compound/91274) |
| Esomeprazole | [9568614](https://pubchem.ncbi.nlm.nih.gov/compound/9568614) |
| CPP-115 | [9794014](https://pubchem.ncbi.nlm.nih.gov/compound/9794014) |
| Ym-298198 | [9819432](https://pubchem.ncbi.nlm.nih.gov/compound/9819432) |
| Ntzdpa | [9954280](https://pubchem.ncbi.nlm.nih.gov/compound/9954280) |
