# Supplementary material for: Biomarker-driven drug repurposing for NAFLD-associated hepatocellular carcinoma using machine learning integrated ensemble feature selection
Source: Front Bioinform. 2025 Apr 17;5:1522401. doi: 10.3389/fbinf.2025.1522401 (PMC12043677; doi:10.3389/fbinf.2025.1522401)
Supplement: Supplementary file 6 [file Table6.docx]

| Protein-ligand complex | The total binding free energy  (dG_Bind) (kcal/mol) | Electrostatic contribution  (dG_Bind_Coulomb) ) (kcal/mol) | Van der Waals interaction energy  (dG_Bind_vdW) ) (kcal/mol) | Lipophilic contribution  (dG_Bind_Lipo) ) (kcal/mol) | Solvation energy components(polar)  (dG_Bind_Solv_GB) ) (kcal/mol) |
| --- | --- | --- | --- | --- | --- |
| ABAT-Esculin | -18.6918101 | -29.43415777 | -8.33270774 | -0.754854018 | 18.5811275 |
| C8B-Diosmin | -74.5784254 | -52.2350777 | -37.39941416 | -34.58605934 | 44.80437799 |
| FBXL3-Phenelzine | -29.6822761 | -16.80022901 | -9.276126427 | -25.56487139 | 19.82120519 |
| ABCB-Diosmin | -89.2674460 | -34.76788482 | -54.00486808 | -40.60457361 | 38.67733718 |
| APOF-Lapatinib | -79.9836040 | -19.4798475 | -48.74298235 | -51.77096205 | 32.96825315 |
| CENPV-Diosmin | -100.705449 | -46.93107614 | -49.49751087 | -47.8595299 | 46.93087651 |
| MBPTS-Diosmin | -80.3378385 | -37.73290748 | -50.39701063 | -41.19207031 | 48.92471385 |
| Zfp1-Diosmin | -69.7496786 | -16.58504309 | -48.13311037 | -41.0425533 | 29.50444272 |
